# Metabolization of Resolvin E4 by ω-Oxidation in Human Neutrophils: Synthesis and Biological Evaluation of 20-Hydroxy-Resolvin E4 (20-OH-RvE4)

**DOI:** 10.1021/acsptsci.3c00201

**Published:** 2023-11-20

**Authors:** Amalie Føreid Reinertsen, Stephania Libreros, Robert Nshimiyimana, Charles Nicholas Serhan, Trond Vidar Hansen

**Affiliations:** † Department of Pharmacy, Section for Pharmaceutical Chemistry, University of Oslo, P.O. Box 1068, 0316 Oslo, Norway; ‡ Center for Experimental Therapeutics and Reperfusion Injury, Department of Anesthesiology, Perioperative and Pain Medicine, Brigham and Women’s Hospital, Harvard Medical School, Boston, Massachusetts 02115, United States

**Keywords:** 20-OH-RvE4, resolvin E4, resolvins, specialized pro-resolving mediators, biosynthesis, omega oxidation

## Abstract

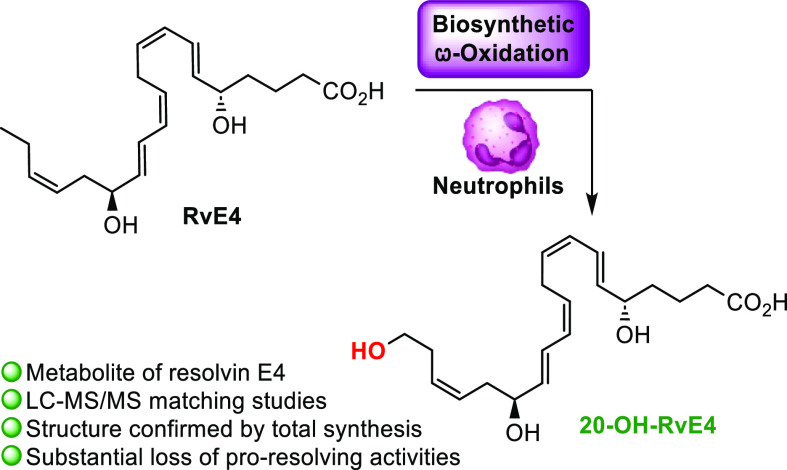

Resolvin E4 (RvE4) belongs to the resolvin family of specialized pro-resolving mediators (SPMs). The resolvins are endogenously formed mediators with both potent pro-resolving and anti-inflammatory biological activities and have attracted considerable attention in both inflammation research and drug discovery. Hence, further metabolism of the resolvins is of interest. Gaining knowledge about the structure–function of further metabolites of the resolvins is important due to their interest in drug-discovery efforts. For the first time, the total synthesis and biological evaluations of the ω-20 hydroxylated metabolite of RvE4, named herein 20-OH-RvE4, are presented. RvE4 was converted to 20-OH-RvE4 by human polymorphonuclear leukocytes. LC–MS/MS analysis and UV spectrophotometry reveal that the synthetic 20-OH-RvE4 matched RvE4-converted product 20-OH-RvE4 by human neutrophils. Cellular studies have revealed that RvE4 is formed from eicosapentaenoic acid in physiologic hypoxia by human neutrophils and macrophages, and we herein established that 20-OH-RvE4 is a secondary metabolite formed by the ω-oxidation of RvE4 in human neutrophils. A direct comparison of the biological actions between RvE4 and its metabolic product suggested that 20-OH-RvE4 displayed reduced bioactions in stimulating the efferocytosis of human senescent erythrocytes by human M2-like macrophages. At concentrations down to 0.1 nM, RvE4 increased macrophage erythrophagocytosis, an important pro-resolving function that was diminished due to metabolic transformation. The results provided herein contribute to a novel molecular insight on the further local metabolization of RvE4, the newest member among the SPM superfamily.

The acute inflammatory response is a pivotal and active immunological process during infection or tissue damage.^1^ Human tissue macrophages are important contributors to the acute inflammation by the biosynthesis of pro-inflammatory lipid mediators like leukotrienes and prostaglandins.^2,3^ Recent efforts have established that the resolution of inflammation is mediated by timely and locally biogenesis and termination programs under the guidance of endogenous mediators coined specialized pro-resolving mediators (SPMs), which encompass the lipoxins, resolvins, protectins, and maresins.^4−8^Figure 1 illustrates the chemical structures of some SPMs and the ω-oxidation metabolite of protectin D1 (PD1), namely, 22-OH-PD1. During the resolution phase of inflammation, the essential ω-3 (docosahexaenoic acid, eicosapentaenoic acid (EPA), and n-3 docosapentaenoic acid) and ω-6 (arachidonic acid) polyunsaturated fatty acids are each converted to specific SPMs mainly by lipoxygenase (LOX) enzymes. SPMs limit the infiltration of neutrophils and increase the removal of apoptotic cells by macrophages.^9,10^ Thus, the active resolution processes of inflammation are orchestrated by SPMs, which are considered a biomedical paradigm shift in thinking and approach to controlling excessive inflammation.^11^

**Figure 1:**
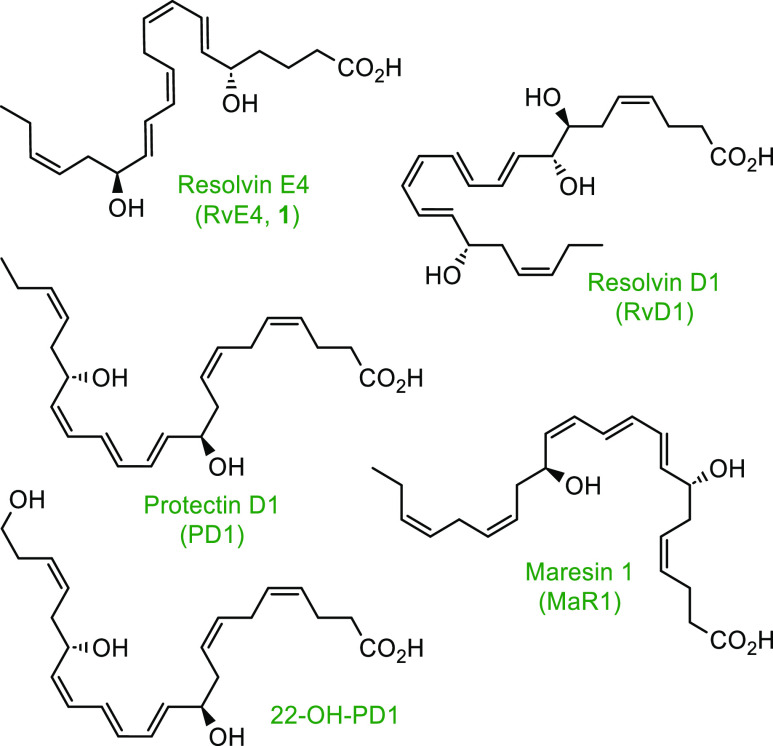
Chemical structures of some SPMs and the ω-oxidation product 22-OH-PD1.

The E-series resolvins are derived from EPA and are among the first SPMs to be elucidated.^12,13^ Recently, the complete stereochemistry of resolvin E4 (RvE4, **1**) and its novel pro-resolving actions were established and confirmed by stereoselective total synthesis.^14,15^ RvE4 (**1**) is produced by human macrophages and neutrophils in physiological hypoxia,^16^ wherein it potently stimulates human macrophage efferocytosis of both senescent red blood cells (sRBCs) (EC_50_ ∼ 0.29 nM) and apoptotic neutrophils (EC_50_ ∼ 0.23 nM).^14,16^ Additionally, RvE4 (**1**) proved to accelerate in vivo the resolution of hemorrhagic exudates in mice by increasing the clearance of apoptotic neutrophils and erythrocytes by macrophage efferocytosis while further limiting neutrophil infiltration at the side of inflammation.^16^Scheme 1 illustrates the proposed biosynthesis of RvE4 (**1**)^16^ and its ω-oxidation product 20-OH-RvE4 (**2**). The first step is initiated by 15-LOX to produce 15*S*-H*p*EPE from EPA, followed by peroxidase activity to reduce the peroxide to the corresponding alcohol 15*S*-HEPE. Next, 15*S*-HEPE is subjected to 5-LOX to form the 15*S*-H,5*S*-H*p*EPE intermediate, which is further subjected to another round of peroxidase enzymes to give RvE4 (**1**). Further metabolism of RvE4 (**1**) is proposed to result in the formation of the carbon 20 position ω-hydroxy metabolite denoted as 20-OH-RvE4 (**2**).

**Scheme 1:**
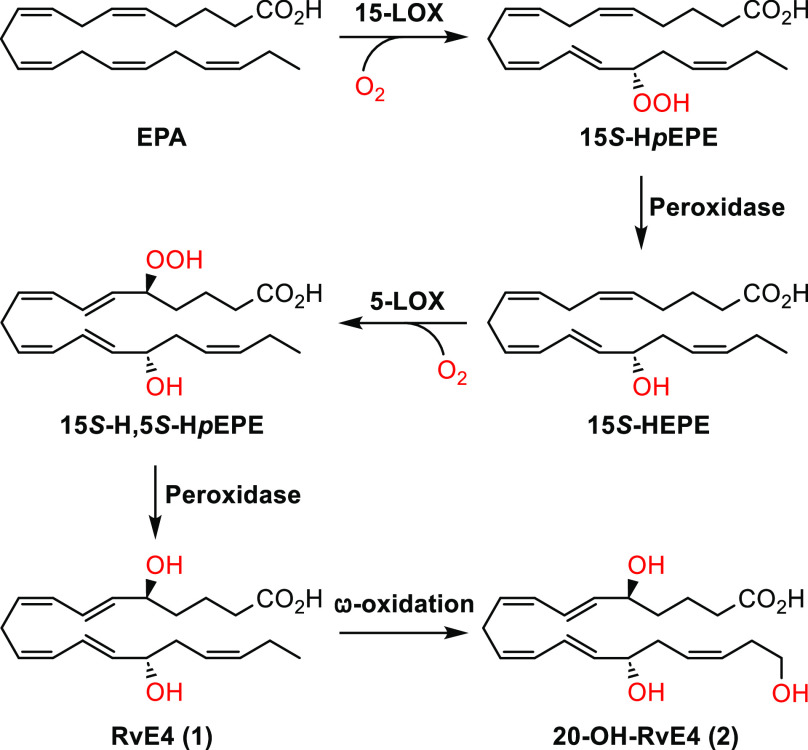
Proposed Biosynthesis of RvE4 (**1**)^16^ and the Putative Formation of 20-OH-RvE4 (**2**)

The SPMs are subjected to oxidative metabolization by both cytochrome (CYP) P450 and eicosaoxidoreductase enzymes.^17−19^ We recently reported the ω-oxidation metabolite of both PD1^20−22^ and its congener, PD1_*n*-3 DPA_.^23^ The ω-oxidation product of PD1 to 22-OH-PD1 (see Figure 1 for the chemical structures) showed potent pro-resolving biological actions in nanomolar concentrations similar to that of PD1.^21^ Herein, we report the stereoselective total synthesis of 20-OH-RvE4 (**2**) and demonstrate its biosynthetic formation from RvE4 (**1**) by human polymorphonuclear neutrophils (PMNs). In addition, we confirm the potent functions of RvE4 (**1**) in increasing macrophage erythrophagocytosis.

## Results and Discussion

**Scheme 2:**
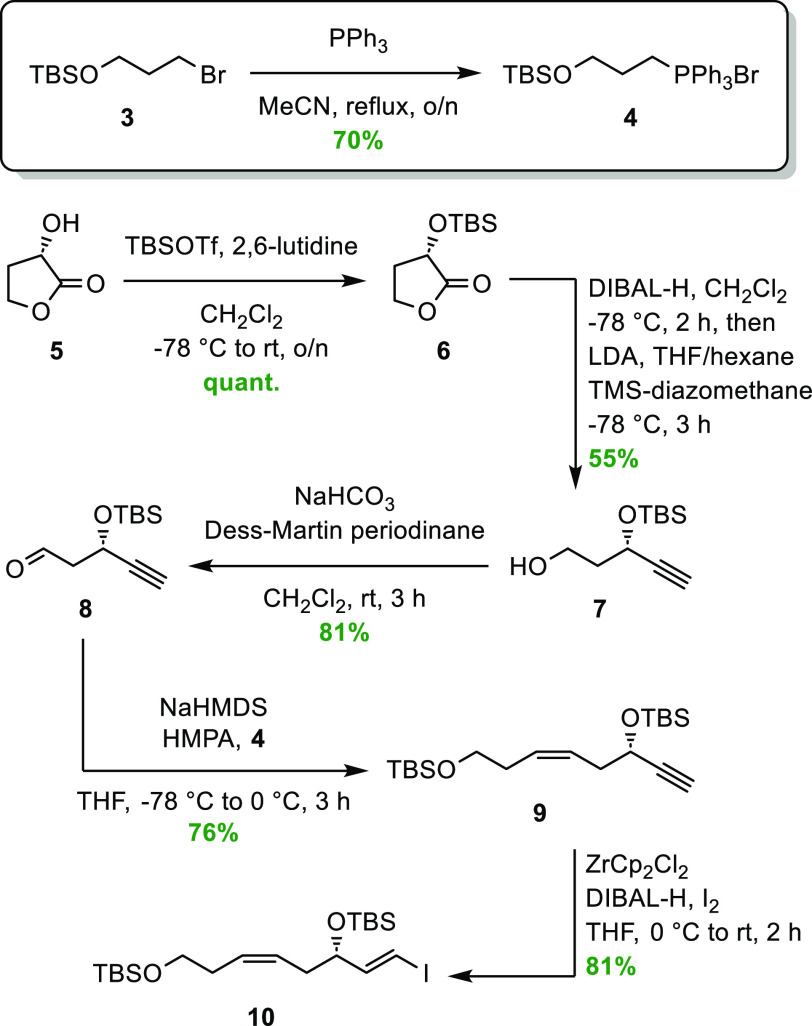
Synthesis of Vinyl Iodide **10**

The stereoselective total synthesis of 20-OH-RvE4 (**2**) commenced with the preparation of vinylic iodide **10** in a five-step sequence starting from (*S*)-(−)-α-hydroxy-γ-butyrolactone (**5**) (Scheme 2). Known aldehyde **8**(24) was reacted in a *Z*-selective Wittig reaction with the ylide of phosphonate **4**. This gave the desired *Z*-olefin (**9**) as previously described in the literature.^22^ Wittig salt **4** was prepared from commercially available (3-bromopropoxy)-*tert*-butyldimethylsilane (**3**), as reported earlier.^25^ A highly *E*-selective hydrozirconation/iodination protocol was applied to transform alkyne **9** into vinyl iodide **10** in a satisfactory 81% yield (*E*/*Z* = 98:2 based on ^1^H NMR analysis).

**Scheme 3:**
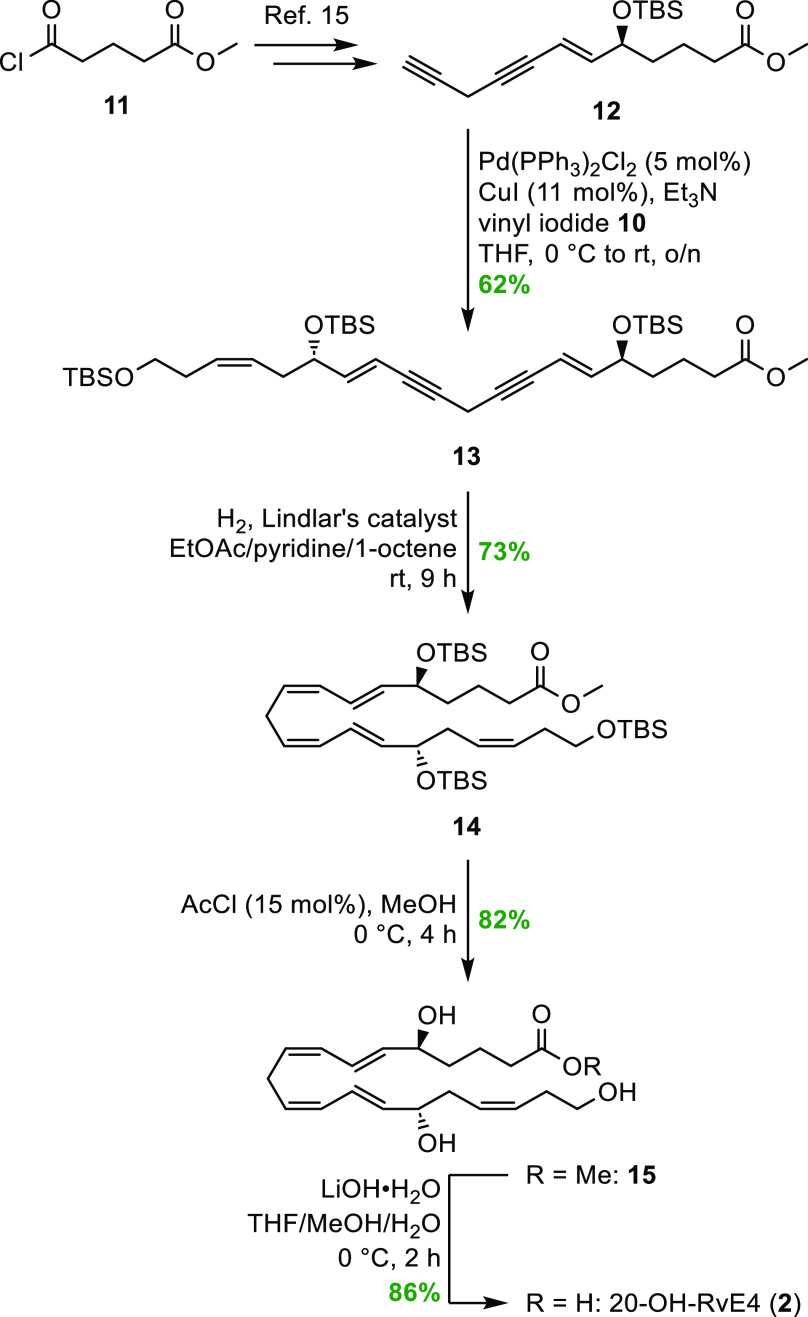
Final Steps to Complete the Stereoselective Synthesis of 20-OH-RvE4 (**2**)

Compound **12** was prepared from commercially available acid chloride **11** in seven steps, as reported earlier by our group for the synthesis of RvE4 (**1**).^15^ By reacting vinyl iodide **10** in a Sonogashira cross-coupling reaction with the highly unstable diyne **12**, compound **13** was obtained in acceptable 62% yield, as shown in Scheme 3. The two triple bonds in compound **13** were then reduced using a Lindlar hydrogenation protocol applying a mixed solvent system containing EtOAc/pyridine/1-octene,^15,24,26−28^ to obtain the desired compound **14**. The pyridine helps reduce the activity of the diverse catalyst, wherein the 1-octene diminishes the tendency of over-reduction. To cleave the three silyl ethers in **14**, catalytic amounts of acetic chloride in MeOH, using dry conditions, afforded the 20-OH-RvE4 methyl ester (**15**) in 82% yield and chemical purity >99% based on high-performance liquid chromatography (HPLC) analysis (Figure S15, Supporting Information). A mild saponification of methyl ester **15** using LiOH·H_2_O in a mixture of THF/MeOH/H_2_O at 0 °C gave the target molecule **2** in 86% isolated yield and chemical purity >99% based on HPLC analysis (Figure S16, Supporting Information). The chemical structure of 20-OH-RvE4 (**2**) was in accord with the obtained NMR, MS, and UV data (Supporting Information).

**Figure 2:**
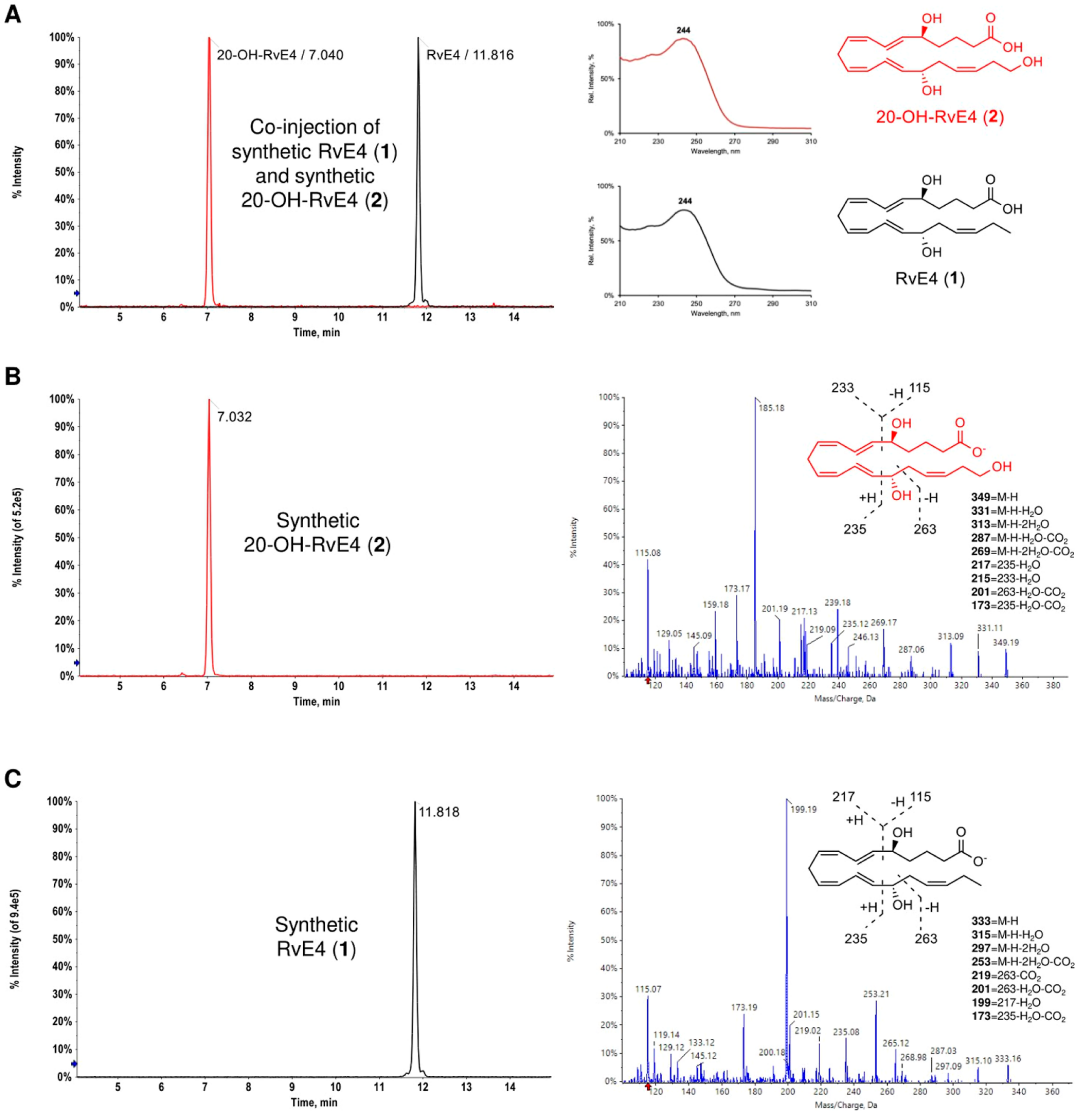
Authentication of synthetic RvE4 (**1**) and 20-OH-RvE4 (**2**) using LC–MS/MS and UV spectrophotometry. (A) *Left*, MRM chromatogram of a coinjection of synthetic 20-OH-RvE4 (**2**, *m*/*z* 349 > 115, red trace) and synthetic RvE4 (**1**, *m*/*z* 333 > 115, black trace). *Right*, UV absorbance spectra of 20-OH-RvE4 (**2**, red) and RvE4 (**1**, black). (B) *Left*, MRM chromatogram of synthetic 20-OH-RvE4 (**2**) alone. *Right*, corresponding MS/MS spectrum of synthetic 20-OH-RvE4 (**2**). (C) *Left*, MRM chromatogram of synthetic RvE4 (**1**) alone. *Right*, corresponding MS/MS spectrum of synthetic RvE4 (**1**).

A direct comparison of synthetic RvE4 (**1**) and synthetic 20-OH-RvE4 (**2**) using LC–MS/MS was first carried out for authentication purposes (Figure 2). Figure 2A shows the multiple reaction monitoring (MRM) chromatogram of a coinjection between synthetic 20-OH-RvE4 (**2**, *m*/*z* 349 > 115, red trace) with a retention time (*T*_r_) = 7.04 min and synthetic RvE4 (**1**, *m*/*z* 333 > 115, black trace) with *T*_r_ = 11.82 min, together with the UV absorbance spectra of **2** (red) and **1** (black). The λ_max_^MeOH^ at 244 nm is characteristic of conjugated diene chromophores present in both molecules.^14−16^Figure 2B depicts the MRM chromatogram of synthetic 20-OH-RvE4 (**2**) together with a MS/MS spectrum showing the molecular ion at *m*/*z* 349 (M – H) as well as the additional daughter ions [*m*/*z* 331 (M – H – H_2_O), *m*/*z* 313 (M – H – 2H_2_O), *m*/*z* 287 (M – H – H_2_O – CO_2_), *m*/*z* 269 (M – H – 2H_2_O – CO_2_), *m*/*z* 235, *m*/*z* 217 (235 – H_2_O), *m*/*z* 215 (233 – H_2_O), *m*/*z* 201 (263 – H_2_O – CO_2_), *m*/*z* 173 (235 – H_2_O – CO_2_), and *m*/*z* 115]. Figure 2C illustrates the MRM chromatogram of synthetic RvE4 (**1**) along with a MS/MS spectrum providing the molecular ion at *m*/*z* 333 (M – H) and the corresponding fragment daughter ions [*m*/*z* 315 (M – H – H_2_O), *m*/*z* 297 (M – H – 2H_2_O), *m*/*z* 253 (M – H – 2H_2_O – CO_2_), *m*/*z* 235, *m*/*z* 219 (263 – CO_2_), *m*/*z* 201 (263 – H_2_O – CO_2_), *m*/*z* 199 (217 – H_2_O), *m*/*z* 173 (235 – H_2_O – CO_2_), and *m*/*z* 115].

**Figure 3:**
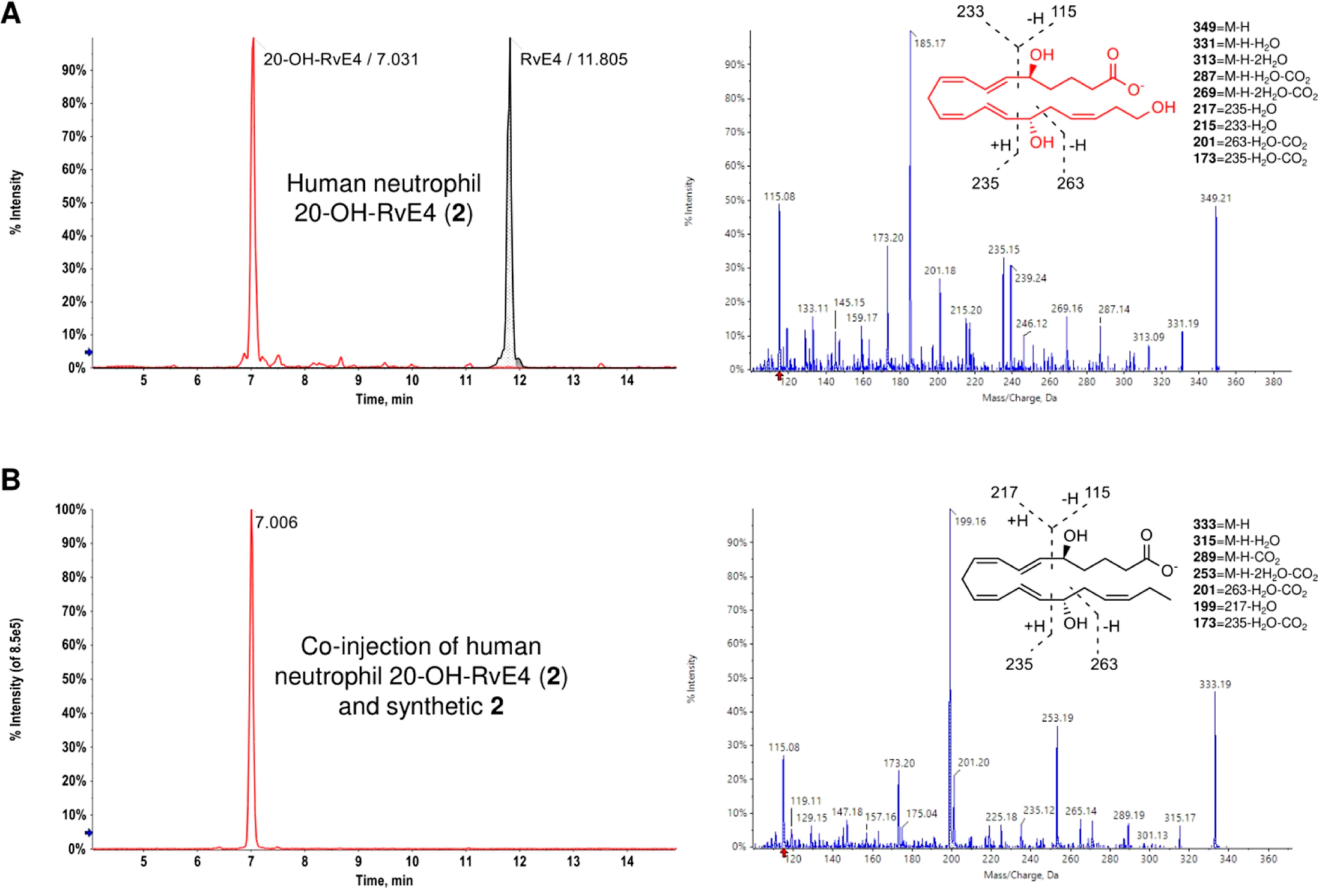
Human PMNs produce the novel RvE4-derived metabolite 20-OH-RvE4 (**2**). (A) *Left*, RvE4 (**1**, 100 ng) was incubated with the isolated human neutrophils (50 × 10^6^ cells in 1.0 mL of Dulbecco’s phosphate-buffered saline (DPBS^+/+^), pH = 7.45, 30 min, 37 °C) to produce 20-OH-RvE4 (**2**). *Right*, MS/MS spectrum of the human neutrophil 20-OH-RvE4 (**2**). (B) *Left*, MRM chromatogram of a coinjection of human neutrophil 20-OH-RvE4 (**2**) and synthetic **2** (*m*/*z* 349 > 115, red trace), ∼150 pg each. *Right*, MS/MS spectrum of RvE4 (**1**) added to the human neutrophils in (A).

The SPMs and their metabolites are biosynthesized in the nano- to picogram scale,^29^ hence the structural authentication of the biogenic products by direct NMR analyses is not possible. To determine whether oxidation at the carbon 20 position represents the foremost route of RvE4 (**1**) further metabolism in human phagocytes, we incubated RvE4 (**1**) with isolated human PMNs (Figure 3). As shown in Figure 3A, left panel, human PMN incubations with synthetic RvE4 (**1**) gave rise to a new product, 20-OH-RvE4 (**2**, red trace), which was identified and eluted at *T*_r_ = 7.03 min, while the synthetic RvE4 (**1**, black shaded trace) eluted at *T*_r_ = 11.81 min. Human neutrophil-derived 20-OH-RvE4 (**2**) revealed a fragmentation pattern with a parent molecular ion at *m*/*z* 349 (M – H) as well as fragment ions [*m*/*z* 331 (M – H – H_2_O), *m*/*z* 313 (M – H – 2H_2_O), *m*/*z* 287 (M – H – H_2_O – CO_2_), *m*/*z* 269 (M – H – 2H_2_O – CO_2_), *m*/*z* 263, *m*/*z* 235, *m*/*z* 233, *m*/*z* 217 (235 – H_2_O), *m*/*z* 215 (233 – H_2_O), *m*/*z* 201 (263 – H_2_O – CO_2_), *m*/*z* 173 (235 – H_2_O – CO_2_), and *m*/*z* 115], as shown in Figure 3A, right panel. Next, we wanted to gain evidence to verify whether coinjections of both compounds (human neutrophil 20-OH-RvE4 (**2**) and synthetic **2**) possessed the same physical characteristics in these same conditions. Coinjection of neutrophil-derived 20-OH-RvE4 (**2**) with synthetic 20-OH-RvE4 (**2**) gave coelution at *T*_r_ = 7.01 min as a single peak (Figure 3B, left panel), thus proving their identical chromatographical behavior. Using a custom spectral library of authentic lipid mediators and SPMs, biogenic 20-OH-RvE4 (**2**) MS/MS spectra matched 99.4% to synthetic 20-OH-RvE4 (**2**). Overall, the above results confirm that the synthetic product of **2** matched the human neutrophil 20-OH-RvE4 (**2**), viz., 5*S*,15*S*,20-trihydroxy-6*E*,8*Z*,11*Z*,13*E*,17*Z*-eicosapentaenoic acid (**2**), thus confirming the complete stereochemistry of 20-OH-RvE4 (**2**). These results demonstrate that 20-OH-RvE4 (**2**) is an oxidation product of RvE4 (**1**) that is produced by human PMNs.

**Figure 4:**
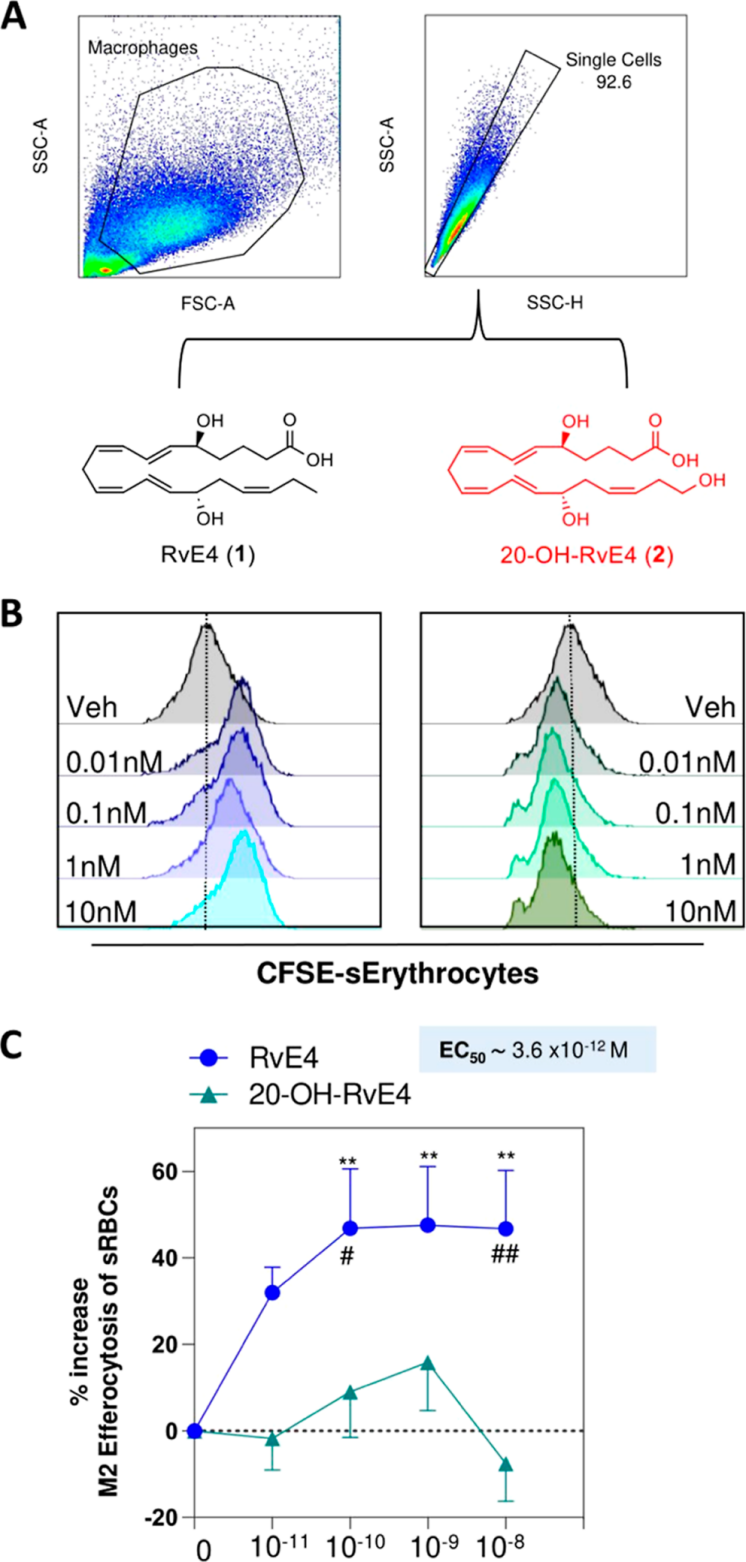
RvE4 (**1**) increases efferocytosis of sRBCs by M2-like macrophages in a dose-dependent manner. (A) Representative flow cytometry gating strategy for M2 macrophages. (B) Representative histogram of M2 intracellular CFSE-labeled sRBCs. For (B), RvE4 (**1**), 20-OH-RvE4 (**2**), and vehicle control were carried out in the same experiments (*n* = 5 separate donors). For clarity, the results were separated for each compound and compared to vehicle alone [i.e., RvE4 (**1**) versus vehicle alone]. The same vehicle representative histogram is presented in all two panels for direct comparisons. (C) Dose–response: Percent increase in M2 efferocytosis of sRBCs above vehicle by RvE4 (**1**) or 20-OH-RvE4 (**2**). The results are represented as mean ± SEM (*n* = 5 healthy human donors). *p < 0.05, **p < 0.01, and ****p < 0.0001 compared to the vehicle control. EC_50_ was estimated using nonlinear regression (dashed line) with log (agonist) versus response (three parameters).

During the resolution of acute inflammation, macrophages play a pivotal role in the removal of cellular debris, apoptotic cells, and senescent cells, a process called efferocytosis.^5^ RvE4 (**1**) is proven to potently stimulate clearance of both apoptotic and aging cells.^16^ We next determined if 20-OH-RvE4 (**2**) retained the potent bioaction of RvE4 (**1**) in regulating the efferocytosis of sRBCs. Isolated peripheral blood mononuclear cells (PBMCs) from human were differentiated and polarized into M2-like human macrophages (see Experimental Section). The M2-like macrophages were chosen due to their anti-inflammatory and repairing mechanisms rather than the pro-inflammatory M1-like macrophages.^30^ The human M2 macrophages were then incubated with 0.01–10 nM of RvE4 (**1**), 20-OH-RvE4 (**2**), or vehicle control (0.01% EtOH) for 15 min, followed by addition of carboxyfluorescein succinimidyl ester (CFSE)-labeled sRBCs (1:50 (macrophages/sRBCs) ratio) for 180 min at 37 °C (Figure 4A) and assayed for erythrophagocytosis by flow cytometry. Representative flow cytometry gating strategy of the M2-like macrophages and histograms of intracellular CFSE-labeled sRBCs from synthetic RvE4 (**1**), synthetic 20-OH-RvE4 (**2**), and vehicle control are reported in Figure 4A,B. RvE4 (**1**) statistically significantly amplified the phagocytosis of senescent erythrocytes in a dose-dependent manner starting at the lowest tested concentration, 0.1 nM, which gave 42.5 ± 14.6% (*p* < 0.01), 1 nM gave 47.6 ± 13.5% (*p* < 0.01), and 10 nM gave 46.7 ± 13.4% (*p* < 0.01) increases when compared to vehicle alone (Figure 4B, blue line). By direct comparison, 20-OH-RvE4 (**2**) did not statistically significantly increase human M2-like macrophage efferocytosis of sRBCs when compared to vehicle alone. We next determined the EC_50_ for each compound; RvE4 (**1**) gave an EC_50_ approximately of 3.6 × 10^–12^ M, while its metabolite 20-OH-RvE4 (**2**) had an EC_50_ of 1.5 × 10^–11^ M (Figure 4C). These results indicate that RvE4 (**1**) was more potent than its further metabolite 20-OH-RvE4 (**2**) in stimulating the efferocytosis of sRBCs by human M2-like macrophages. Along these lines, the conversion of RvE4 (**1**) to its 20-OH-product metabolite (**2**) represents an inactivation pathway for RvE4 (**1**).

**Figure 5:**
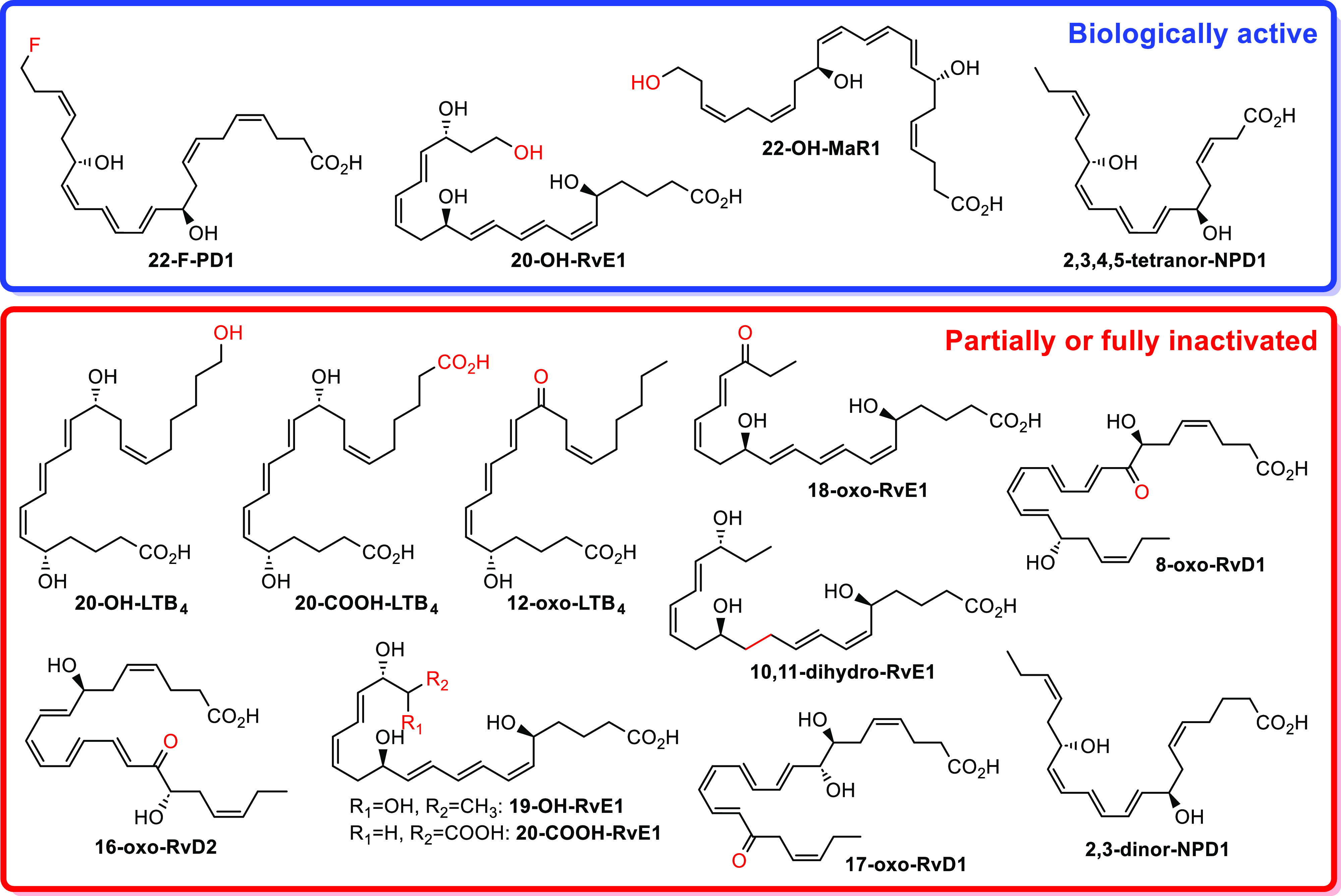
Structures of some further metabolites of the SPMs and the pro-inflammatory mediator LTB_4_. Also, the chemical structure of the biologically active synthetic analogue 22-F-PD1 is given.

Autacoids are physiologically active substances that are produced by the body and typically have a localized effect and a short duration of action. In many cases, local ω-oxidation reactions give a decrease in the bioactivity of the local autacoids, e.g., the pro-inflammatory lipid mediator LTB_4_. LTB_4_ is metabolized by 12-hydroxydehydrogenase to give the inactive 12-oxo-LTB_4_^31^ or by CYP450 enzymes to give the considerably less potent 20-OH-LTB_4_,^32^ as illustrated in Figure 5. Prostaglandin E_2_ (PGE_2_) is inactivated by 15-prostaglandin dehydrogenase to 15-oxo-PGE_2_.^33^ Also, RvE1, LXA_4_, RvD1, and RvD2 are substrates for 15-prostaglandin dehydrogenase enzymes.^8,34−36^ Metabolism of some SPMs may give products that retain the anti-inflammatory and pro-resolving actions of the original SPM, e.g., 20-OH-RvE1, 22-OH-PD1, and 22-OH-MaR1 (see Figures 1 and 5 for the chemical structures). The ω-oxidation metabolite of RvE1, namely, 20-OH-RvE1, proved to be essentially as potent as its parent SPM (i.e., RvE1) in reducing the infiltration of neutrophils into inflamed peritonea.^36^ Similar to RvE1, 20-OH-RvE1 stops PMN infiltration,^36^ and thus, it is possible that 20-OH-RvE4 (**2**) is also bioactive with other cell types. However, our original discoveries of RvE4 (**1**) as a potent bioactive mediator were based on its ability to stimulate the efferocytosis of senescent erythrocytes as described in Norris et al.^16^ 22-OH-PD1 limits leukocyte recruitment in the same extent as PD1,^21,37^ while 22-OH-MaR1 was found to display similar potencies as the parent SPM at regulating human macrophage responses to *Escherichia coli* (*E. coli*).^38^ Of note, the synthetic analogue 22-F-PD1 (Figure 5) also showed potent pro-resolving and anti-inflammatory properties in a dose-dependent manner in the low nanomolar range.^22^ In other cases, the metabolism of SPMs can give a partially or fully loss of the biological activity, as shown for 16-oxo-RvD2,^35^ 19-OH-RvE1, 10,11-dihydro-RvE1, 18-oxo-RvE1, 20-COOH-RvE1,^36^ 8-oxo-RvD1, and 17-oxo-RvD1^34,35^ (see Figure 5 for the chemical structures). A highly interesting study on the metabolization of PD1 by β-oxidation of its polar head chain found that 2,3,4,5-tetranor-NPD1 (C18 metabolite), but not 2,3-dinor-NPD1 (C20 metabolite), maintained the anti-inflammatory and pro-resolving bioactivities of the parent SPM (Figure 5).^39^ The results presented herein suggest that the conversion of RvE4 (**1**) to 20-OH-RvE4 (**2**) by human neutrophils is an inactivation route for the potent and novel resolvin, RvE4 (**1**). Further inactivation of 20-OH-RvE4 (**2**) may include transformations to give the dicarboxylate, as reported for RvE1 and LTB_4_ respective metabolites, 20-OH-RvE1 and 20-OH-LTB_4_, that give the partially or fully inactivated 20-COOH-RvE1^36^ and 20-COOH-LTB_4_ (Figure 5).^32,40^ If RvE4 (**1**) displays this or other pathways of metabolization (e.g., oxidation of the two chiral allylic alcohols) and whether this indeed is an inactivation route remain to be investigated in future studies.

## Conclusions

The first total synthesis of 20-OH-RvE4 (**2**), an ω-oxidation metabolite of the novel SPM RvE4 (**1**), has been described in 9% overall yield over 11 synthetic steps, thus establishing the exact configuration of the biomolecule 20-OH-RvE4 (**2**). We also present for the first time the conversion of RvE4 (**1**) to the ω-hydroxy further metabolite 20-OH-RvE4 (**2**) produced by human neutrophils. The ω-oxidation of RvE4 (**1**) to 20-OH-RvE4 (**2**) is most likely mediated by CYP1 monooxygenases, as reported for some other SPMs.^18,19,37^ RvE4 (**1**) is a potent resolution agonist in that it decreases leukocyte recruitment in vivo.^16^ 20-OH-RvE4 (**2**) showed a considerable loss of activity compared to RvE4 (**1**) in stimulating human macrophage efferocytosis of sRBCs. In contrast, the ω-oxidation of RvE1, PD1, and MaR1 to 20-OH-RvE1, 22-OH-PD1, and 22-OH-MaR1, respectively, provided bioactive products with similar potencies as the parent SPM.^21,36,38^ However, if 20-OH-RvE4 (**2**) has actions comparable to RvE4 (**1**) on other target cell types remain to be investigated. Overall, the results presented herein specify important information about the local further metabolization of the resolvins and are of great interest toward future development of new anti-inflammatory and pro-resolving lead candidates in drug discovery.

## Experimental Section

### (*S*,*Z*)-5-((*E*)-2-Iodovinyl)-2,2,3,3,12,12,13,13-octamethyl-4,11-dioxa-3,12-disilatetradec-7-ene (**10**)

Alkyne **9** was prepared as previously described in the literature studies.^22,24^ Bis(cyclopentadienyl)zirconium(IV) dichloride (194 mg, 0.663 mmol, 1.50 equiv) was suspended in dry THF (1.50 mL) and cooled to 0 °C. Diisobutylaluminum hydride (1 M in THF, 0.66 mL, 0.663 mmol, 1.50 equiv) was added dropwise, and the resulting mixture was stirred for 30 min. Alkyne **9** (163 mg, 0.442 mmol, 1.00 equiv) was dissolved in dry THF (0.25 mL) and added in a dropwise manner at 0 °C. The suspension was warmed to RT and stirred until completion determined by thin-layer chromatography (TLC) (∼1 h) and then cooled back to 0 °C. Iodine (146 mg, 0.575 mmol, 1.30 equiv) was dissolved in dry THF (0.70 mL) and added dropwise at 0 °C. The cooling bath was removed, and stirring was continued at RT for another 40 min. After completion, sat. Na_2_S_2_O_3_ (2 mL), NaHCO_3_ (2 mL), H_2_O (2 mL), and Et_2_O (5 mL) were added, and the phases were separated. The aq. phase was extracted with Et_2_O (3 × 5 mL), and the combined organic phase was dried over Na_2_SO_4_ and filtered, and the solvent was removed by reduced pressure. Purification by flash chromatography (SiO_2_, 1% EtOAc in heptane) afforded vinyl iodide **10** (178 mg, 0.358 mmol, 81%, *E*/*Z* = 98:2 based on ^1^H NMR) as a clear oil. *R*_*f*_ (5% EtOAc in heptane) = 0.67; [α]_*D*_^20^ = −120.6 (*c* 0.10, benzene); ^1^H NMR (400 MHz, CDCl_3_): δ 6.53 (dd, *J* = 14.4, 5.7 Hz, 1H), 6.21 (dd, *J* = 14.4, 1.3 Hz, 1H), 5.54–5.39 (m, 2H), 4.10 (qd, *J* = 6.2, 1.3 Hz, 1H), 3.60 (t, *J* = 7.0 Hz, 2H), 2.28–2.23 (m, 4H), 0.89 (2 × s, 18H), 0.06 (s, 6H), 0.05 (s, 3H), 0.03 (s, 3H); ^13^C NMR (101 MHz, CDCl_3_): δ 148.8, 128.6, 126.3, 76.0, 75.1, 63.0, 35.9, 31.5, 26.1 (3C), 26.0 (3C), 18.5, 18.4, −4.5, −4.7, −5.1 (2C); HRESIMS *m*/*z*: 519.1582 [M + Na]^+^ (calcd for C_20_H_41_INaO_2_Si_2_, 519.1582).

### Methyl (5*S*,6*E*,13*E*,15*S*,17*Z*)-5,15,19-tris((*tert*-Butyldimethylsilyl)oxy)nonadeca-6,13,17-trien-8,11-diynoate (**13**)

Alkyne **12** was prepared as reported in the literature.^15^ Vinyl iodide **10** (40 mg, 81 μmol, 1.00 equiv), dissolved in dry THF (600 μL), was cooled to 0 °C in an ice bath. CuI (1.7 mg, 8.90 μmol, 11 mol %), Pd(PPh_3_)_2_Cl_2_ (2.8 mg, 4.0 μmol, 5 mol %), and Et_3_N (16.3 mg, 22 μL, 0.161 mmol, 2.00 equiv) were added in a successive manner. Alkyne **12** (33 mg, 97 μmol, 1.20 equiv), dissolved in dry THF (600 μL), was added in a dropwise manner, and the resulting suspension was warmed to RT and stirred overnight while protected from light. The suspension was filtered through a short silica plug (15% EtOAc in heptane), and the solvent was removed by reduced pressure. The coupled product **13** (35 mg, 49.8 μmol, 62%) was obtained as a clear oil after purification by flash chromatography (SiO_2_, 2.5% EtOAc in heptane). *R*_*f*_ (10% EtOAc in heptane) = 0.30; [α]_*D*_^20^ = +20.7 (*c* 0.1, benzene); ^1^H NMR (400 MHz, CDCl_3_): δ 6.09 (ddd, *J* = 15.8, 12.5, 5.3 Hz, 2H), 5.64 (ddq, *J* = 15.9, 3.9, 2.0 Hz, 2H), 5.51–5.40 (m, 2H), 4.19–4.14 (m, 2H), 3.66 (s, 3H), 3.60 (t, *J* = 7.0 Hz, 2H), 3.42 (t, *J* = 2.2 Hz, 2H), 2.30 (t, *J* = 7.4 Hz, 2H), 2.28–2.21 (m, 4H), 1.68–1.61 (m, 2H), 1.53–1.47 (m, 2H), 0.89 (2 × s, 27H), 0,07 (s, 3H), 0.05 (s, 3H), 0.04 (s, 6H), 0.03 (s, 3H), 0.02 (s, 3H); ^13^C NMR (101 MHz, CDCl_3_): δ 174.1, 146.1, 146.0, 128.2, 126.8, 109.0, 108.7, 83.8, 83.6, 79.2, 79.0, 72.5, 72.2, 62.9, 51.6, 37.2, 36.2, 34.1, 31.5, 26.1 (3C), 26.0 (6C), 20.5, 18.5, 18.4, 18.3, 11.3, −4.4 (2C), −4.7, −4.8, −5.1 (2C); HRESIMS *m*/*z*: 725.4414 [M + Na]^+^ (calcd for C_39_H_70_NaO_5_Si_3_, 725.4423).

### Methyl (5*S*,6*E*,8*Z*,11*Z*,13*E*,15*S*,17*Z*)-5,15,20-tris((*tert*-Butyldimethylsilyl)oxy)eicosa-6,8,11,13,17-pentaenoate (**14**)

Compound **13** (8.0 mg, 11 μmol, 1.0 equiv), dissolved in EtOAc/pyridine/1-octene (10:1:1, 0.2 mL), was added Lindlar’s catalyst (5.0 mg). The flask was evacuated and refilled with the hydrogen gas three times. More Lindlar’s catalyst (5.0 mg) was added after 2 h. The suspension was stirred and followed by TLC analysis (the semireduced intermediate is observed just above the starting material, and the product can be detected just above the semireduced intermediate using 12% EtOAc in heptane as the eluent system). After completion (∼9 h), the suspension was filtered through a silica plug (15% EtOAc in heptane), and the solvent was removed by reduced pressure. Compound **14** (5.7 mg, 8 μmol, 73%) was isolated as a clear oil after purification by flash chromatography (SiO_2_, 1% EtOAc in heptane). *R*_*f*_ (12% EtOAc in heptane) = 0.48; [α]_*D*_^20^ = +4.6 (*c* 0.13, CH_2_Cl_2_); ^1^H NMR (400 MHz, CDCl_3_): δ 6.45 (dddt, *J* = 15.2, 11.1, 6.4, 1.2 Hz, 2H), 6.02–5.96 (m, 2H), 5.66 (ddd, *J* = 15.1, 13.3, 6.1 Hz, 2H), 5.48–5.46 (m, 2H), 5.35 (dtd, *J* = 11.2, 7.5, 3.7 Hz, 2H), 4.22–4.16 (m, 2H), 3.66 (s, 3H), 3.59 (t, *J* = 7.0 Hz, 2H), 3.05 (tt, *J* = 7.5, 1.7 Hz, 2H), 2.32 (t, *J* = 7.4 Hz, 2H), 2.31–2.22 (m, 4H), 1.74–1.60 (m, 2H), 1.55–1.49 (m, 2H), 0.90 (2 × s, 18H), 0.89 (s, 9H), 0.6 (2 × s, 6H), 0.05 (s, 6H), 0.04 (s, 3H), 0.03 (s, 3H); ^13^C NMR (101 MHz, CDCl_3_): δ 174.2, 137.2 (2C), 129.3, 129.2, 128.7, 128.6, 127.7, 127.4, 124.6, 124.4, 73.1, 72.9, 63.0, 51.6, 37.8, 36.7, 34.2, 31.5, 26.6, 26.1 (3C), 26.0 (6C), 20.9, 18.5, 18.4 (2C), −4.1, −4.2, −4.5, −4.6, −5.1 (2C); HRESIMS *m*/*z* 729.4738 [M + Na]^+^ (calcd for C_39_H_74_NaO_5_Si_3_, 729.4736).

### Methyl (5*S*,6*E*,8*Z*,11*Z*,13*E*,15*S*,17*Z*)-5,15,20-Trihydroxyeicosa-6,8,11,13,17-pentaenoate (**15**)

Compound **14** (12 mg, 17 μmol, 1.00 equiv) was azeotroped twice with 2-Me-THF (0.8 mL) before it was cooled to 0 °C. A premade solution of newly distilled acetic chloride in dry MeOH (0.13 mL, 2.6 μmol, 15 mol %) was added dropwise. After completion determined by TLC (∼4 h), CH_2_Cl_2_ (0.3 mL) was added, followed by H_2_O (0.2 mL) and a 10% aq. solution of NaHCO_3_ (20 μL). The phases were separated, and the aqueous phase was extracted with CH_2_Cl_2_ (3 × 0.3 mL). The combined organic phase was dried (Na_2_SO_4_) and filtered, and the solvent was removed under reduced pressure. Purification by flash chromatography (SiO_2_, 2.5% MeOH in CH_2_Cl_2_) afforded 20-OH-RvE4 methyl ester (**15**, 5.1 mg, 14 μmol, 82%) as a clear oil. The chemical purity (>99%) was determined by HPLC analysis (Eclipse XDB-C18, MeOH/H_2_O 65:35, 1.0 mL/min): *T*_r_(minor) = 6.68, 10.55, and 11.41 min and *T*_r_(major) = 8.83 min. *R*_*f*_ (5% MeOH in CH_2_Cl_2_) = 0.23; [α]_*D*_^20^ = +10.0 (*c* 0.20, MeOH); UV–vis (MeOH) λ_max_ 243 nm (log ε = 4.80); ^1^H NMR (400 MHz, CD_3_OD): δ 6.58 (ddd, *J* = 15.2, 11.0, 1.2 Hz, 2H), 6.01 (td, *J* = 10.6, 1.9 Hz, 2H), 5.69 (td, *J* = 14.9, 6.5, Hz, 2H), 5.57–5.48 (m, 2H), 5.39 (dt, *J* = 11.1, 8.0 Hz, 2H), 4.14 (ddd, *J* = 19.0, 12.9, 6.5 Hz, 2H), 3.66 (s, 3H), 3.55 (t, *J* = 6.8 Hz, 2H), 3.10 (tt, *J* = 7.6, 1.7 Hz, 2H), 2.36 (t, *J* = 7.3 Hz, 2H), 2.35–2.27 (m, 4H), 1.76–1.58 (m, 2H), 1.57–1.49 (m, 2H); ^13^C NMR (101 MHz, CD_3_OD): δ 175.8, 137.8, 137.5, 130.3 (2C), 129.6 (2C), 129.0, 128.4, 126.3 (2C), 73.0, 72.8, 62.6, 52.0, 37.7, 36.4, 34.6, 31.9, 27.4, 22.1; HRESIMS *m*/*z*: 387.2141 [M + Na]^+^ (calcd for C_21_H_32_NaO_5_, 387.2142).

### 5*S*,15*S*,20-Trihydroxy-6*E*,8*Z*,11*Z*,13*E*,17*Z*-eicosapentaenoic Acid (**2**)

Methyl ester **15** (2.5 mg, 6.9 μmol, 1.0 equiv) was dissolved in THF/MeOH/H_2_O (2:2:1, 0.84 mL) and cooled to 0 °C. Solid LiOH·H_2_O (8.8 mg, 0.21 mmol, 31 equiv) was added. The mixture was stirred at this temperature for 3 h, warmed to RT, stirred for an additional 5 min before it was cooled back to 0 °C, and diluted with EtOAc (1.5 mL). Sat. aq. NaH_2_PO_4_ (1.0 mL) was added, the layers were separated, and the aq. layer was extracted with EtOAc (3 × 1.5 mL). The organic layer was dried over Na_2_SO_4_ and filtered, and the solvent was removed by reduced pressure. Purification by flash chromatography (SiO_2_, 10% MeOH in CH_2_Cl_2_) afforded 20-OH-RvE4 free acid (**2**, 1.9 mg, 5.9 μmol, 86%) as a clear oil. The chemical purity (>99%) was established by HPLC analysis (Eclipse XDB-C18, MeOH/3.3 mM aq. AcOH 55:45, 1.0 mL/min): *T*_r_(minor) = 14.63 and 15.98 min and *T*_r_(major) = 16.92 min. *R*_*f*_ (10% MeOH in CH_2_Cl_2_) = 0.12; [α]_*D*_^20^ = +47.9 (*c* 0.020, MeOH); UV–vis (MeOH) λ_max_ 243 nm (log ε = 4.80); ^1^H NMR (600 MHz, CD_3_OD): δ 6.58 (ddd, *J* = 15.2, 11.1, 1.1 Hz, 2H), 6.01 (td, *J* = 10.6, 4.3 Hz, 2H), 5.69 (td, *J* = 15.0, 6.5 Hz, 2H), 5.56–5.50 (m, 2H), 5.41–5.36 (m, 2H), 4.17 (qd, *J* = 6.4, 1.2, 1H), 4.13 (q, *J* = 6.5 Hz, 1H), 3.55 (t, *J* = 6.8 Hz, 2H), 3.10 (tt, *J* = 7.6, 1.6 Hz, 2H), 2.37–2.28 (m, 4H), 2.29 (t, *J* = 7.2 Hz, 2H), 1.74–1.60 (m, 2H), 1.60–1.52 (m, 2H); ^13^C NMR (151 MHz, CD_3_OD): δ 178.6, 137.9, 137.5, 130.3, 130.2, 129.6 (2C), 129.0, 128.4, 126.3 (2C), 73.0, 72.9, 62.6, 37.9, 36.4, 35.6, 31.9, 27.4, 22.5; HRESIMS *m*/*z*: 349.2020 [M – H]^−^ (calcd for C_20_H_29_O_5_, 349.2020).

#### Neutrophil Incubations and Lipid Mediator Metabololipidomics

Human neutrophils (50 × 10^6^) were incubated with RvE4 (**1**, 100 ng/mL, 0.3 μM, from Cayman Chemicals) for 30 min at 37 °C in DPBS^+/+^ (pH = 7.45). Termination of the incubations was performed by adding two portions of frosty LC–MS-grade MeOH (Thermo Fisher Scientific, Waltham, MA) comprising deuterated internal standard *d*_4_-LTB_4_ and *d*_5_-RvD3 purchased from the Cayman Chemical Company (Ann Arbor, MI). The samples were put at −80 °C for a minimum of 30 min to enable protein precipitation, followed by centrifugation (3000 rpm, 10 min, 4 °C). The supernatants were gathered before concentrated to approximately 1 mL using a calm stream of nitrogen (Turbo Vap LV, Biotage, Charlotte, NC). Solid-phase extraction (SPE) was next carried out using an automated system on an Extrahera (Biotage, Charlotte, NC).^41^

Briefly, the samples were brought to an apparent pH = 3.5 using 9 mL of acidified H_2_O and were rapidly loaded onto C18 ISOLUTE 100 mg SPE cartridges (Biotage, Charlotte, NC), which were preconditioned with 3 mL of MeOH and 3 mL of double-distilled H_2_O. The samples were then neutralized with 4 mL of double-distilled H_2_O, followed by a 4 mL hexane (Supelco, Bellefonte, PA) wash. The lipid mediators were eluted in 4 mL of formic acid methyl ester (Sigma-Aldrich, St. Louis, MO). The formic acid methyl ester fractions were concentrated under a calm flow of nitrogen (Turbo Vap LV, Biotage) followed by immediate resuspension in 50 μL of a LC–MS-grade MeOH–H_2_O mixture (1:1, *v*/*v*) for LC–MS/MS data acquisition.

Data were acquired in negative polarity on a Triple Quadrupole 7500 mass spectrometer (SCIEX, Framingham, MA) coupled with a SCIEX ExionLC system and a Kinetex PS C18 100 Å column 100 mm × 3.0 mm × 2.6 μm (Phenomenex, Torrance, CA) maintained at 50 °C. The mobile phase comprised Solvent A (H_2_O, 0.1% formic acid) and Solvent B (MeOH, 0.1% formic acid). RvE4 (**1**) and 20-OH-RvE4 (**2**) were eluted using a 0.5 mL/min flow rate in a gradient of Solvent A/Solvent B (55/45, *v*/*v*) from 0 to 2 min. The second segment was changed to Solvent A/Solvent B (20/80, *v*/*v*) from 2 to 16.5 min, and the third segment was increased to Solvent A/Solvent B (2/98, *v*/*v*) from 16.6 to 18.5 min. The final segment consisted of Solvent A/Solvent B (90/10, *v*/*v*) from 18.6 to 20.9 min.

Source and gas parameters were set as follows: collision gas = 12, curtain gas = 40, ion source gas 1 (psi) = 45, ion source gas 2 (psi) = 70, ion spray voltage (V) = −2000, and temperature (°C) = 500. Data were obtained by using SCIEX OS 3.1.5.3945 and analyzed with SCIEX OS 3.1.5.3945. Identification of 20-OH-RvE4 (**2**) was accomplished by matching both its chromatographic retention time and tandem mass spectral data to its synthetic version. A customized MS/MS library containing spectra of the synthetic material was utilized to assess the spectral fit score. Spectral parameters were set as follows: precursor mass tolerance ±0.8 Da; collision energy ±5 eV; use polarity, intensity threshold = 0.05; minimal purity = 5.0%; and intensity factor = 100. Note that the accuracy for mass spectral data acquisition of the SCIEX 7500 is ±0.1 atomic mass units (a.m.u.). The additional digits presented in the spectral data are due to default settings. UV spectra were obtained on a Cary 3500 Compact Peltier UV–vis Spectrophotometer (Agilent Technologies, Santa Clara, CA). LC–ESI–MS/MS data are presented as screen captures taken from the SCIEX software.

#### Human PMN Isolation

Fresh human blood was collected with heparin (10 U/mL) from healthy volunteers, as approved by the Mass General Brigham Institutional Review Board (protocol 1999P001297). PMNs were isolated by density gradient using Ficoll histopaque (Sigma-Aldrich, 10771). PMN cell purity and cell viability (98 ± 0.5%) were assessed by flow cytometry.

#### Human M2-like Macrophage Polarization and Differentiation

Human PBMCs were obtained from deidentified leukopacks from Boston Children’s Hospital Blood Bank (Boston, MA) under protocol 1999P001279 approved by the Mass General Brigham Institutional Review Board. The peripheral mononuclear cells were isolated by a Ficoll-Histopaque-1077 density gradient, followed by isolation of monocytes by adhesion. Monocytes were differentiated for 7 days in RPMI 1640 (Lonza) containing 10% fetal calf serum (Thermo Fisher Scientific, 16000-044), 2 mM l-glutamine (Lonza, 17-605E), 2 mM penicillin–streptomycin (Lonza, 17-602E), and 20 ng/mL of recombinant human macrophage colony-stimulating factor (hr-MCSF) (PeproTech, 300-25) at 37 °C. Macrophages were then polarized into M2 macrophages with 20 ng/mL of IL-4 (PeproTech, 200-04) for 48 h.^14,16^

#### Human Erythrocyte Cell Isolation and Human M2 Erythrophagocytosis

Human peripheral blood erythrocytes from healthy human volunteers were isolated by centrifugation and aspiration of the platelet-rich plasma and the buffy coat layer. RBCs were purified by resuspension in phosphate-buffered saline (PBS, Lonza, NJ) (10% hematocrit) and centrifugation (500*g*) followed by aspiration of the top 10% of the erythrocyte layer (this purification procedure was carried out with six repetitions). Purified RBCs were then resuspended in PBS (20% hematocrit) and kept at 4 °C for over 96 h to induce aging. sRBCs were counted, washed 2 times with DPBS (Thermo Fisher Scientific, MA), and then stained with Cell TraceTM CFSE at a concentration of 5 μM for 30 min at 37 °C (Thermo Fisher Scientific, MA).

Human M2-like macrophages were seeded into six-well plates at a density of 2 × 10^6^ cells per well in DPBS containing Ca^2+^ and Mg^2+^. Cells were incubated with either RvE4 (**1**), 20-OH-RvE4 (**2**) (0.01, 0.1, 1, or 10 nM), or vehicle alone (containing 0.01% EtOH *v*/*v*) for 15 min at 37 °C before the addition of CFSE-labeled sRBCs [1:50 (M2 macrophages/sRBCs) ratio]. After 3 h at 37 °C, cells were washed 6 times with DPBS containing 5 mM ethylenediaminetetraacetic acid (EDTA) for the removal of undigested and membrane-bound sRBCs. M2 macrophages were detached from plates using EDTA (5 mM) and fixed in fluorescence-activated cell sorting buffer containing 2% paraformaldehyde (Electron Microscopy Sciences). CFSE fluorescence associated with efferocytosis was measured by using flow cytometry. All flow cytometric samples were measured using a BD LSR-Fortessa (BD Biosciences, CA) and analyzed using a FlowJO X.

## Supporting Information Available

^1^H NMR, ^13^C NMR, and HRMS data of **10**, **13**, **14**, **15**, and **2**; COSY NMR, UV–vis, and HPLC data of **15** and **2** (PDF)

## Abbreviations

CFSE: carboxyfluorescein succinimidyl ester
CYP450: cytochrome P450
DPBS: Dulbecco’s phosphate-buffered saline
EDTA: ethylenediaminetetraacetic acid
LOX: lipoxygenase
MRM: multiple reaction monitoring
PBMC: peripheral blood mononuclear cells
PBS: phosphate buffered saline
PMN: polymorphonuclear neutrophil
sRBCs: senescent red blood cells
