# Supplementary material for: Metabolization of Resolvin E4 by ω-Oxidation in Human Neutrophils: Synthesis and Biological Evaluation of 20-Hydroxy-Resolvin E4 (20-OH-RvE4)
Source: ACS Pharmacol Transl Sci. 2023 Nov 20;6(12):1898–908. doi: 10.1021/acsptsci.3c00201 (PMC10714428; doi:10.1021/acsptsci.3c00201)
Supplement: Supplementary file 1 — pt3c00201_si_001.pdf [file pt3c00201_si_001.pdf]

Metabolization of Resolvin E4 by ω-Oxidation in Human Neutrophils: Synthesis and Biological Evaluation of 20-Hydroxy-Resolvin E4 (20-OH-RvE4)

Amalie Føreid Reinertsen,[†*] *Stephania Libreros,*[§] *Robert Nshimiyimana,*[§] *Charles Nicholas Serhan,*[§] *and Trond Vidar Hansen*[†]

†Department of Pharmacy, Section for Pharmaceutical Chemistry, University of Oslo, P.O. Box 1068, 0316 Oslo, Norway

§Center for Experimental Therapeutics and Reperfusion Injury, Department of Anesthesiology, Perioperative and Pain Medicine, Brigham and Women's Hospital, Harvard Medical School, Boston, MA

*For correspondence: Amalie Føreid Reinertsen, a.f.reinertsen@farmasi.uio.no

Table of contents

General information……………………………………………………………….S-2

^{1}H-, ^{13}C-, and COSY NMR Spectra……………………………………………….S-3

UV-Vis Spectra…………………………………………………………………….S-9

HPLC Chromatograms…………………………………………………………….S-10

HRMS Spectra……………………………………………………………………..S-12

General information. All commercially available reagents and solvents were used in the form they were supplied without any further purification, unless otherwise indicated. The stated yields are based on isolated material. All reactions were performed under an argon atmosphere using Schlenk techniques, unless stated otherwise. Reaction flasks were covered with aluminum foil during sensitive reactions and storage to minimize exposure to light. Thin layer chromatography was performed on silica gel 60 F254 aluminum-backed plates fabricated by Merck. Flash column chromatography was performed on silica gel 60 (40-63 μm) produced by Merck. NMR spectra were recorded on a Bruker AVII400 spectrometer at 400 MHz for ^{1}H NMR and at 101 MHz for ^{13}C NMR or a Bruker AVII600 spectrometer at 600 MHz for ^{1}H NMR and at 151 MHz for ^{13}C NMR. Coupling constants (J) are reported in hertz and chemical shifts are reported in parts per million (δ) relative to the central residual protium solvent resonance in ^{1}H NMR ($CDCl_3$ = δ 7.26 and CD_3OD = δ 3.31) and the central carbon solvent resonance in ^{13}C NMR ($CDCl_3$ = δ 77.00 ppm and CD_3OD = δ 49.00). Optical rotations were measured using a Perkin Elmer 341 polarimeter. Mass spectra were recorded at 70 eV on Micromass Prospec Q or Micromass QTOF 2 W spectrometer using ESI as the method of ionization. High-resolution mass spectra were recorded at 70 eV on Micromass Prospec Q or Micromass QTOF 2W spectrometer using ESI as the method of ionization. HPLC-analyses were performed using a C_{18} stationary phase (Eclipse XDBC$_{18}$, 4.6 x 250 mm, particle size 5 μm, from Agilent Technologies), applying the conditions stated. The UV-Vis spectra were recorded using an Agilent Technologies Cary 8485 UV-VIS spectrophotometer using quartz cuvettes.

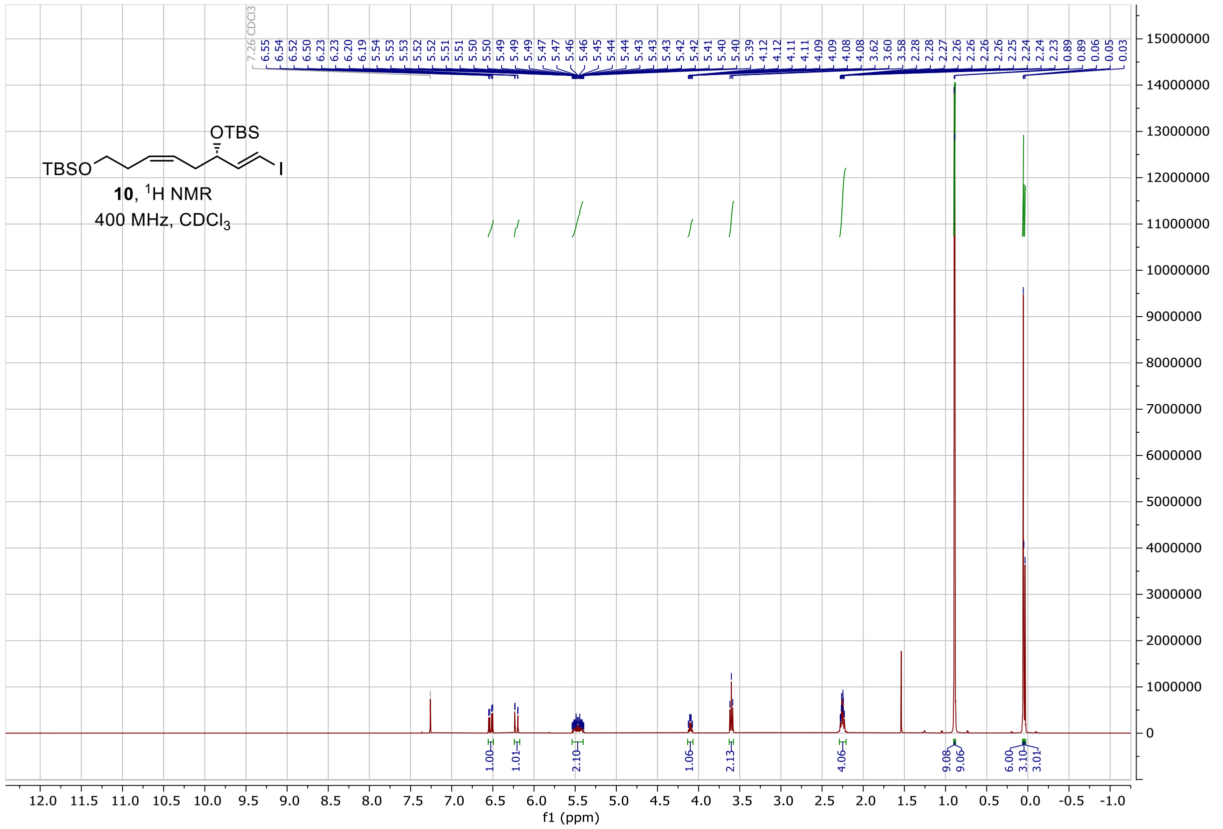

Figure S1 ^{1}H NMR spectrum of vinyl iodide **10**.

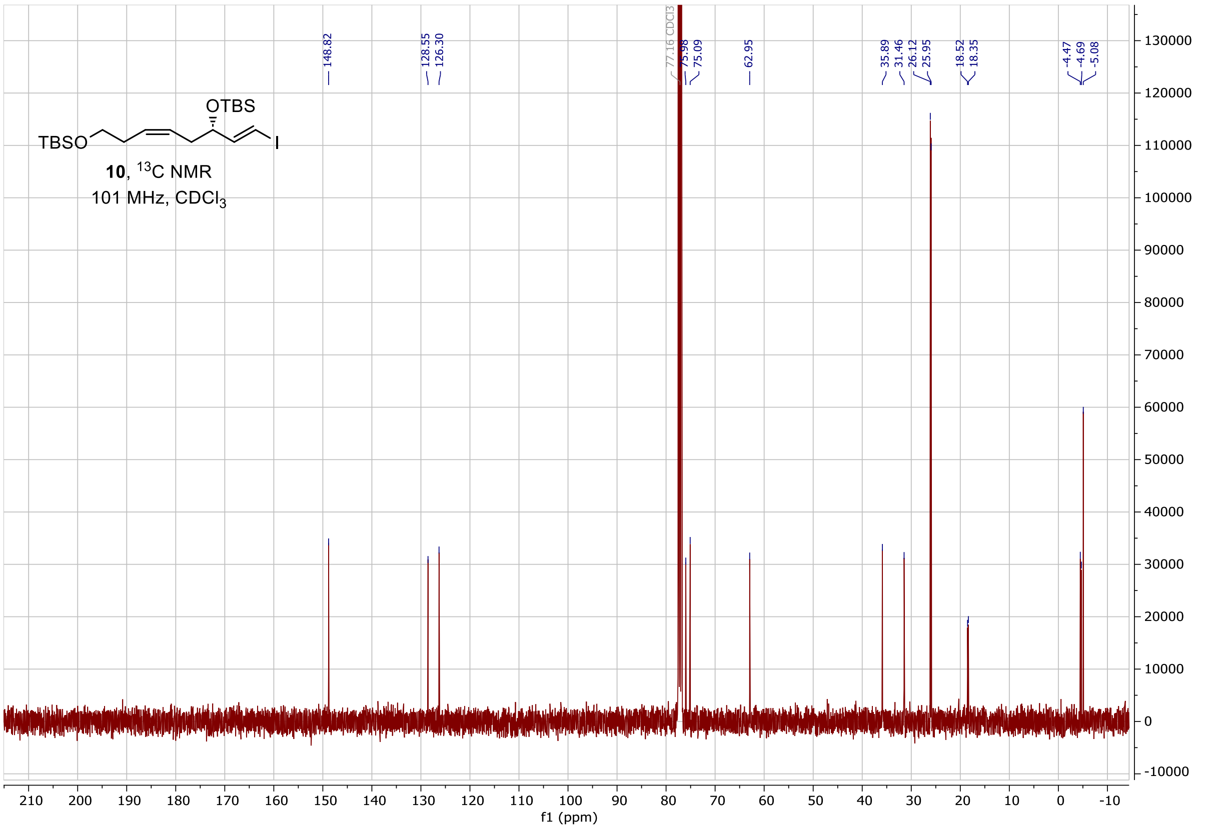

Figure S2 ^{13}C NMR spectrum of vinyl iodide **10**.

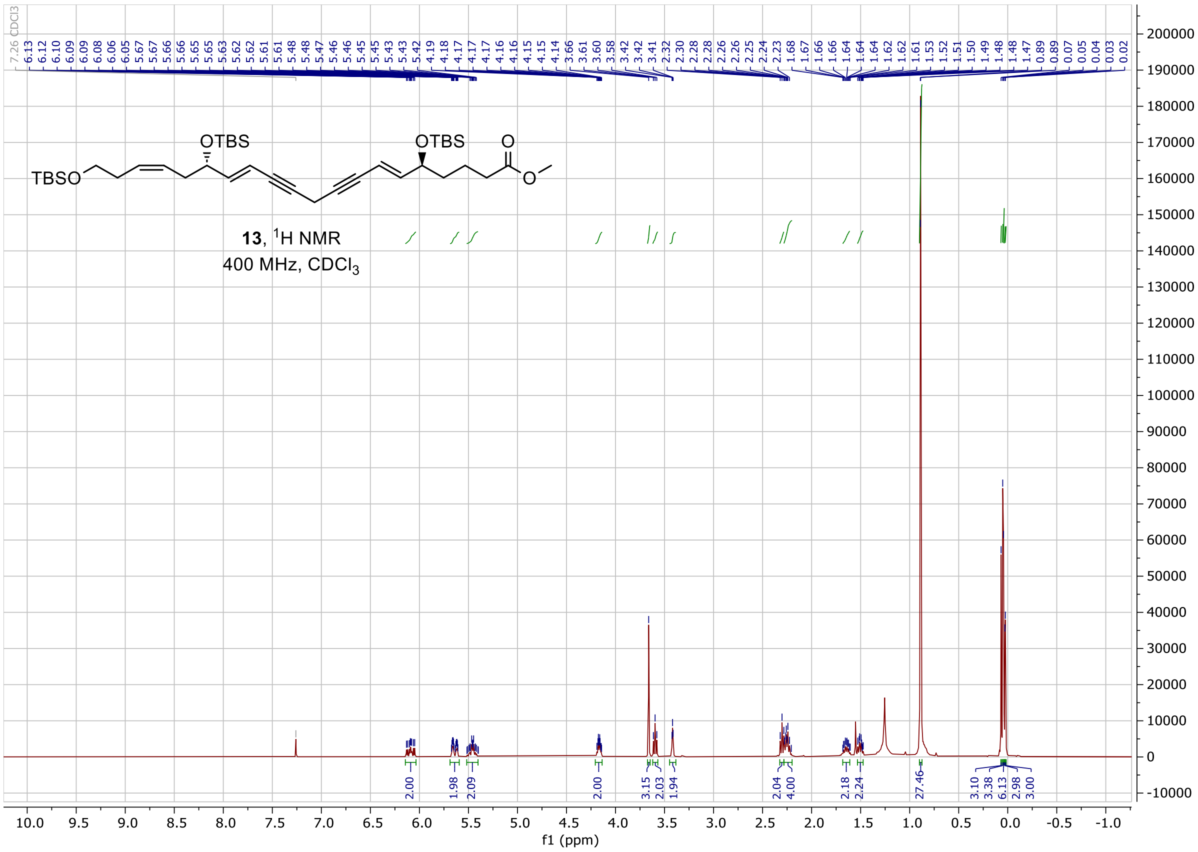

Figure S3 ^{1}H NMR spectrum of diyne **13**.

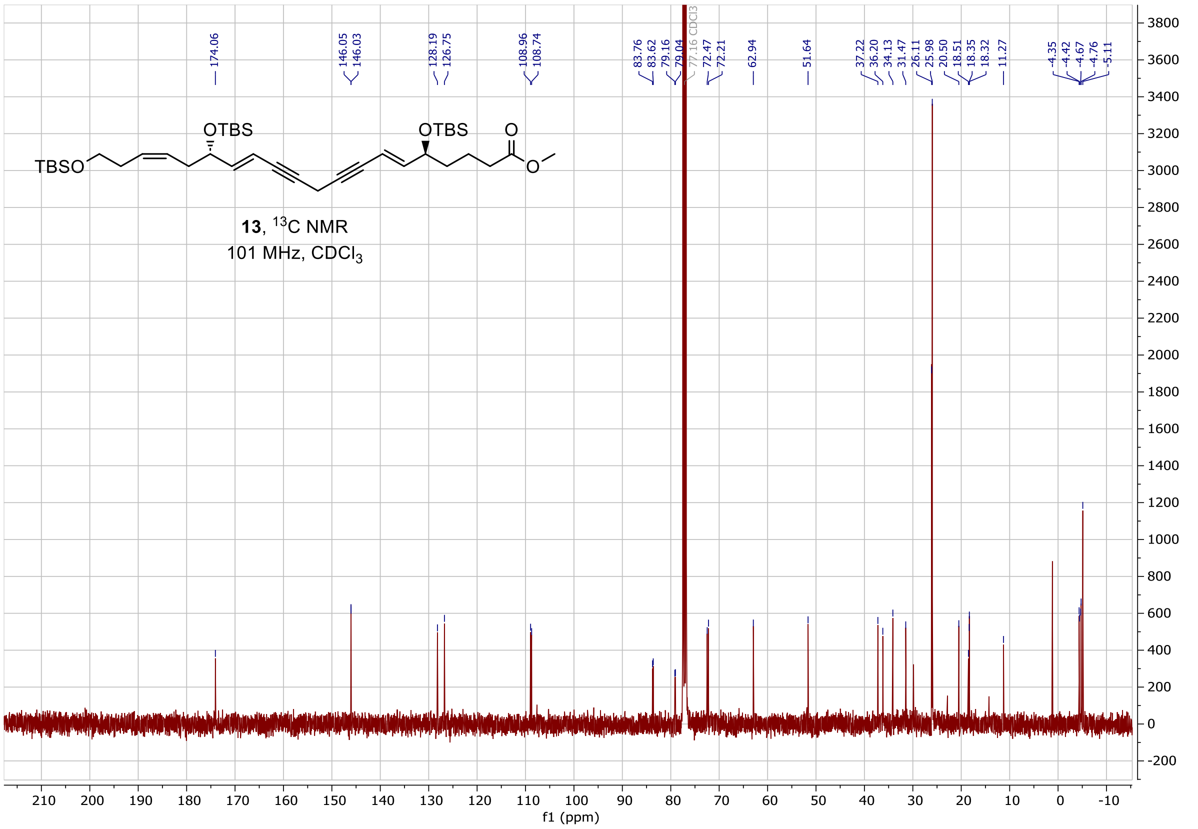

Figure S4 ^{13}C NMR spectrum of diyne **13**.

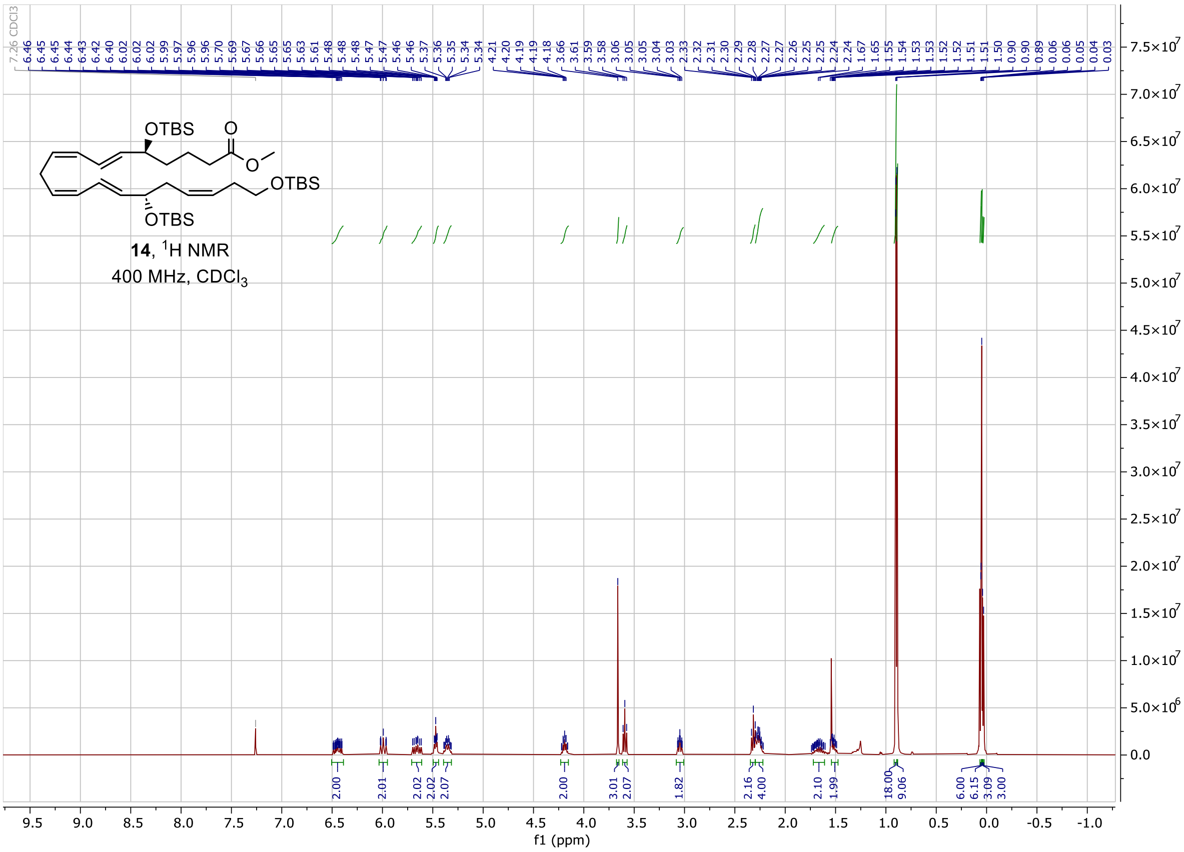

Figure S5 ^{1}H NMR spectrum of compound **14**.

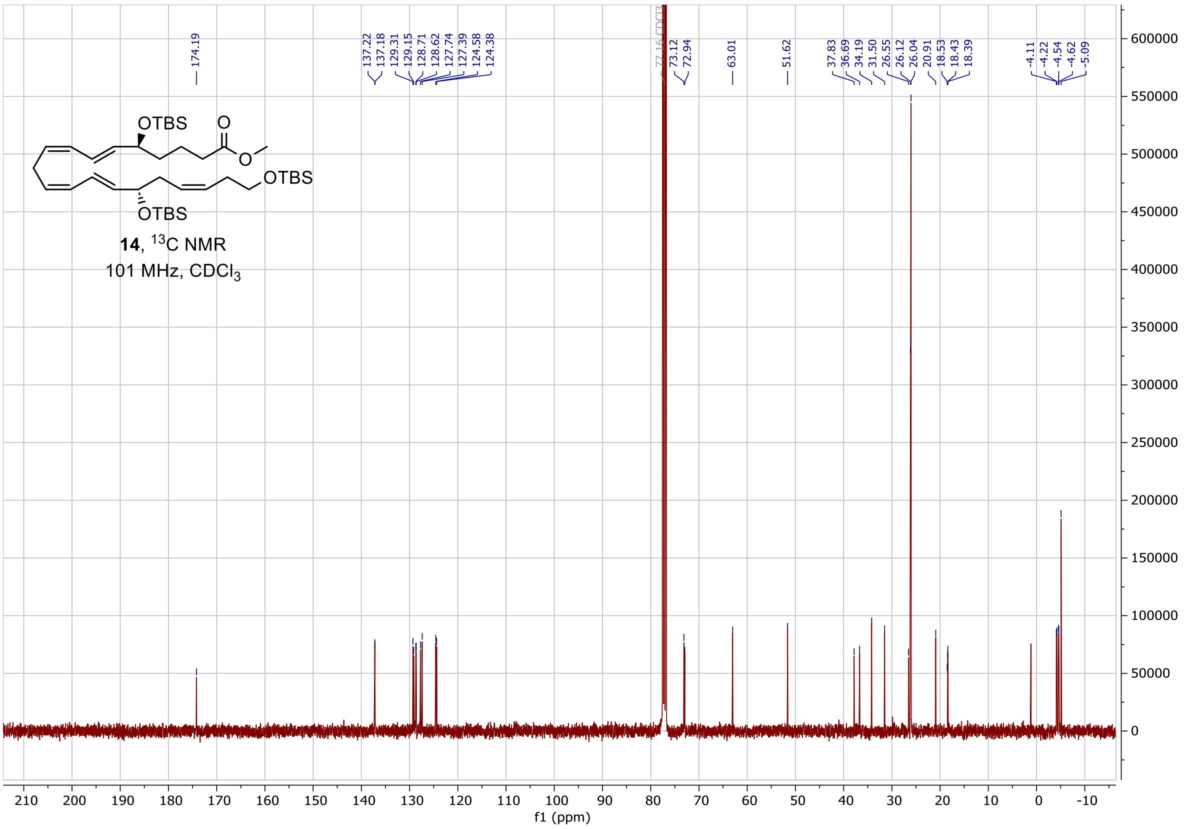

Figure S6 ^{13}C NMR spectrum of compound **14**.

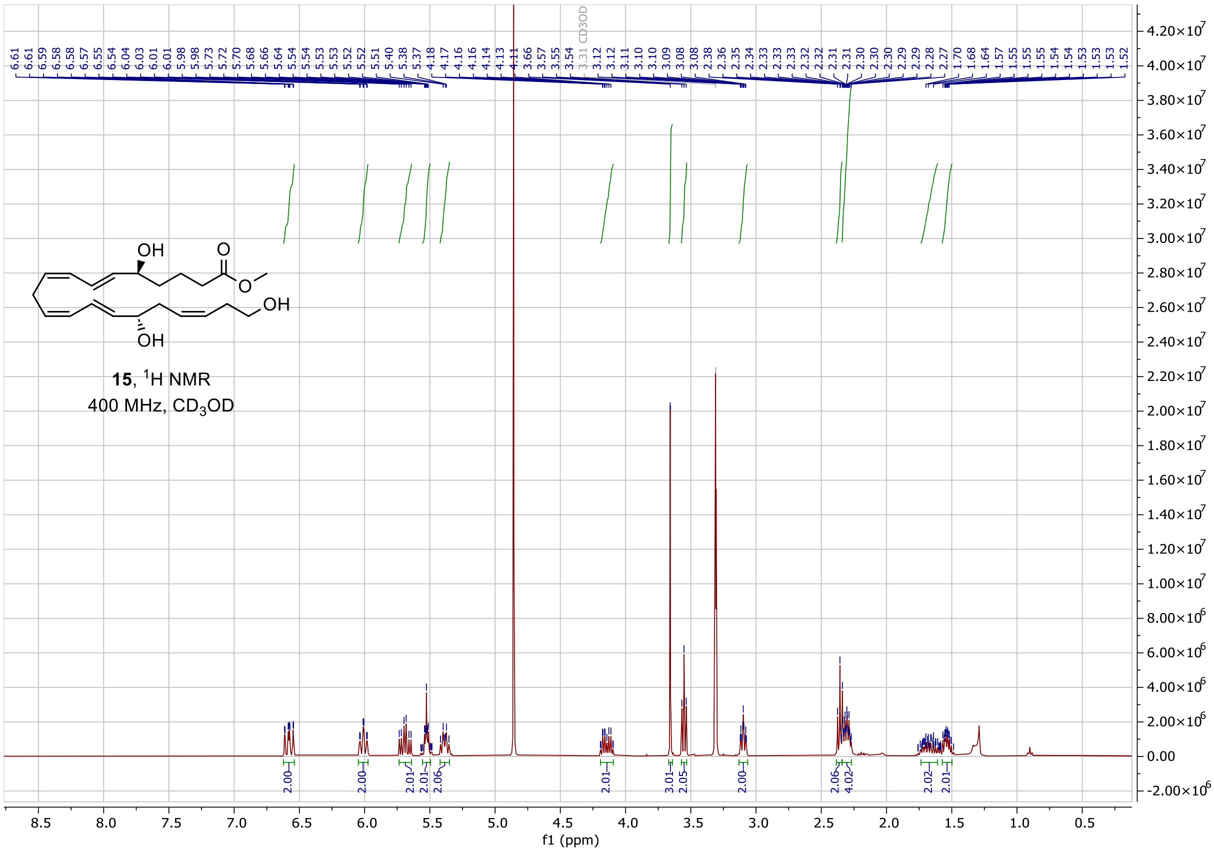

Figure S7 ^{1}H NMR spectrum of 20-OH-RvE4 methyl ester (**15**).

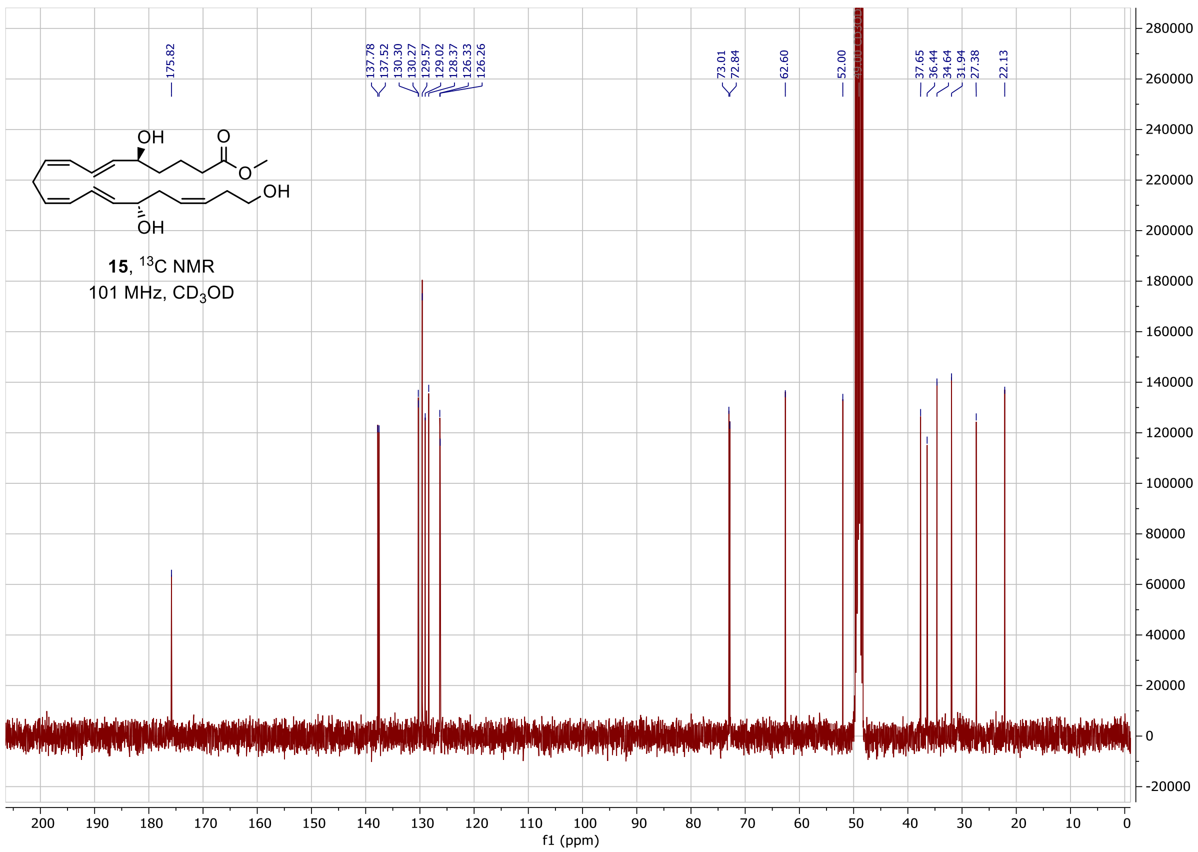

Figure S8 ^{13}C NMR spectrum of 20-OH-RvE4 methyl ester (**15**).

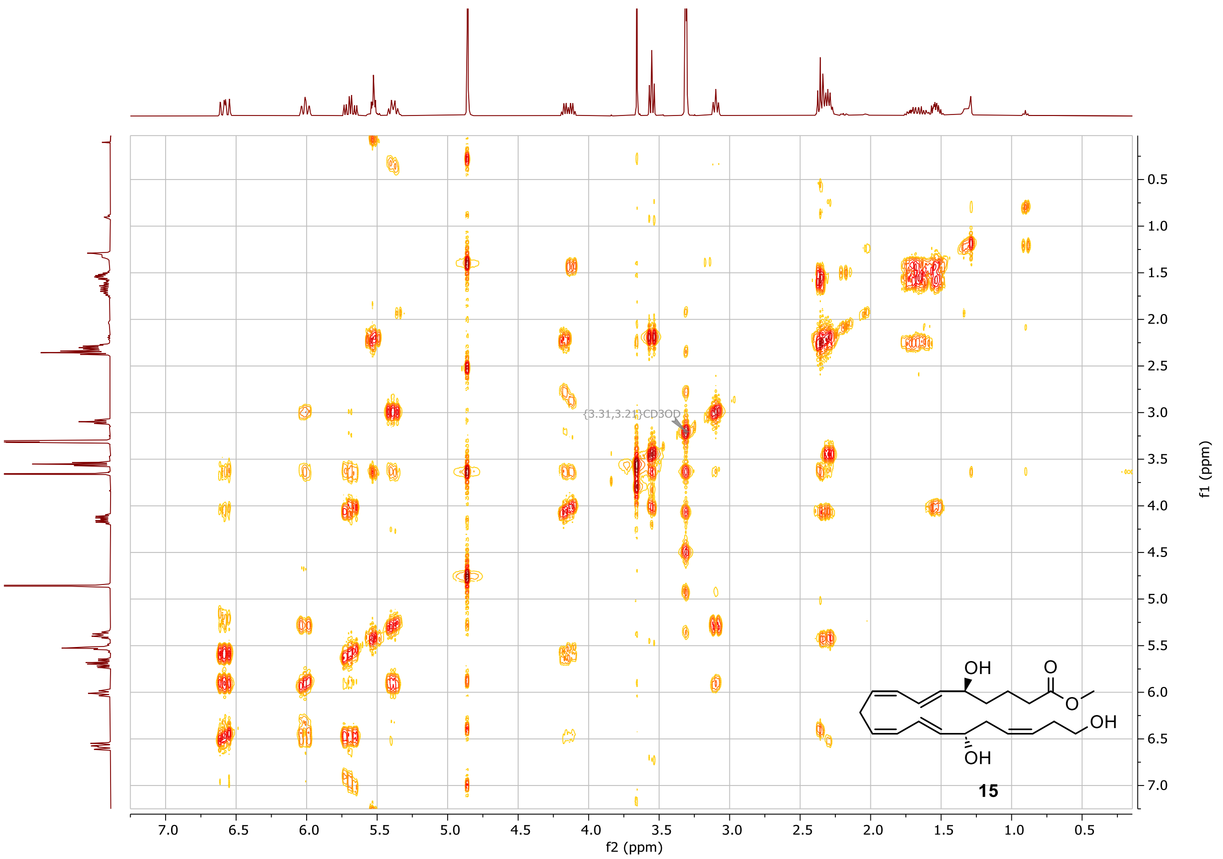

Figure S9 COSY NMR spectrum of 20-OH-RvE4 methyl ester (**15**).

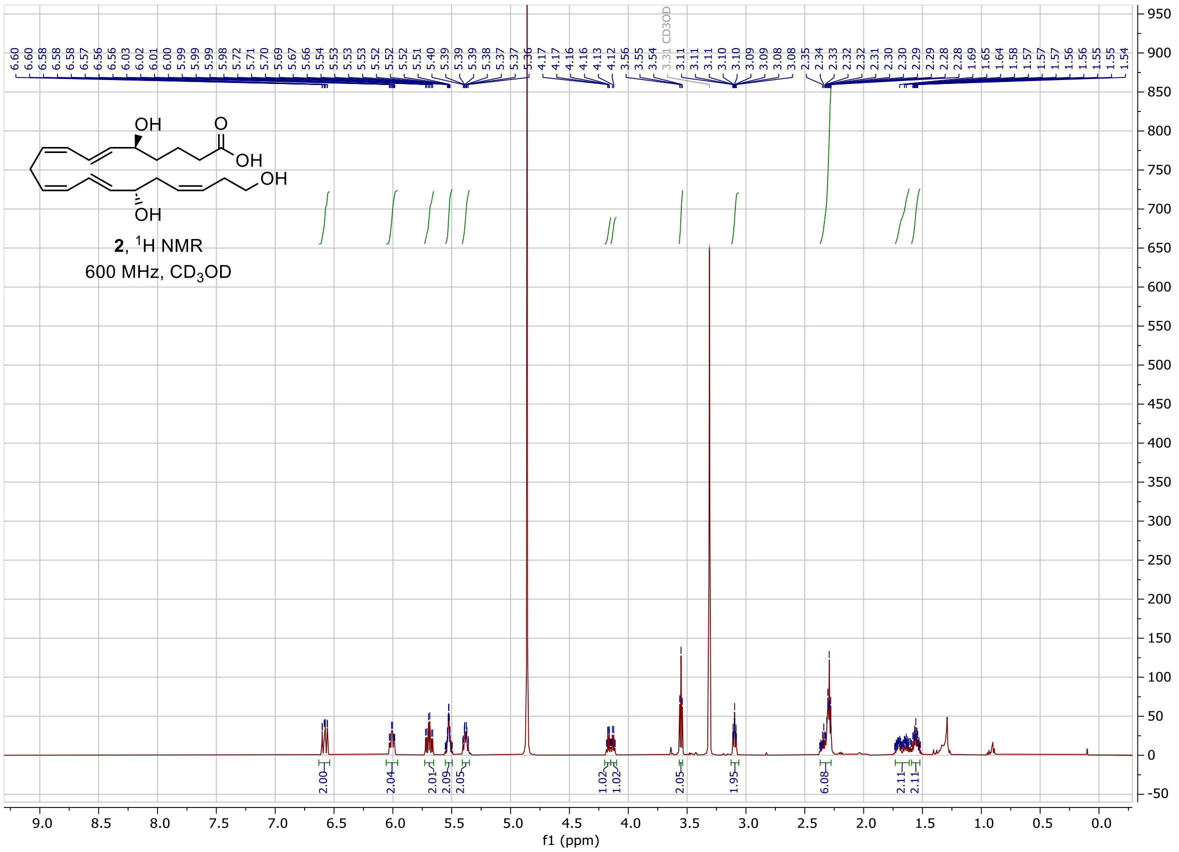

Figure S10 ^{1}H NMR spectrum of 20-OH-RvE4 (**2**).

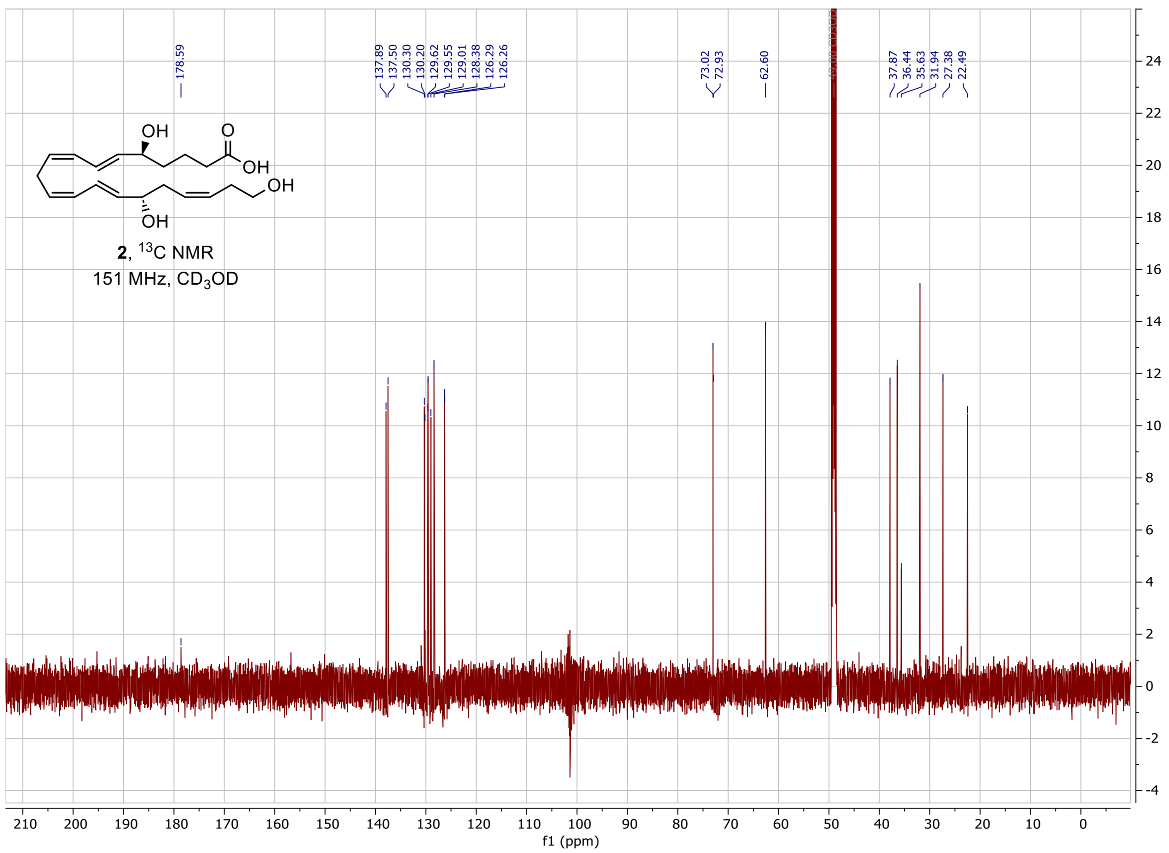

Figure S11 ^{13}C NMR spectrum of 20-OH-RvE4 (**2**).

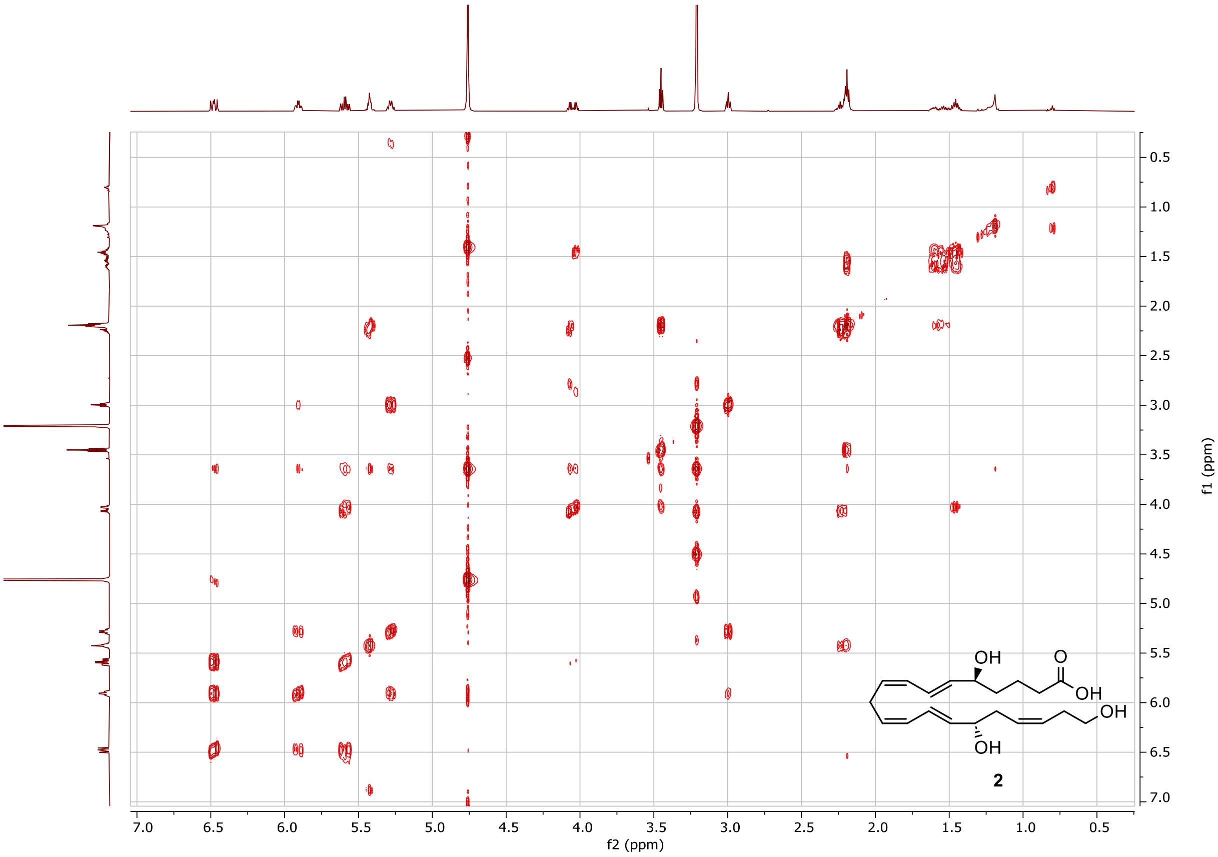

Figure S12 COSY NMR spectrum of 20-OH-RvE4 (**2**).

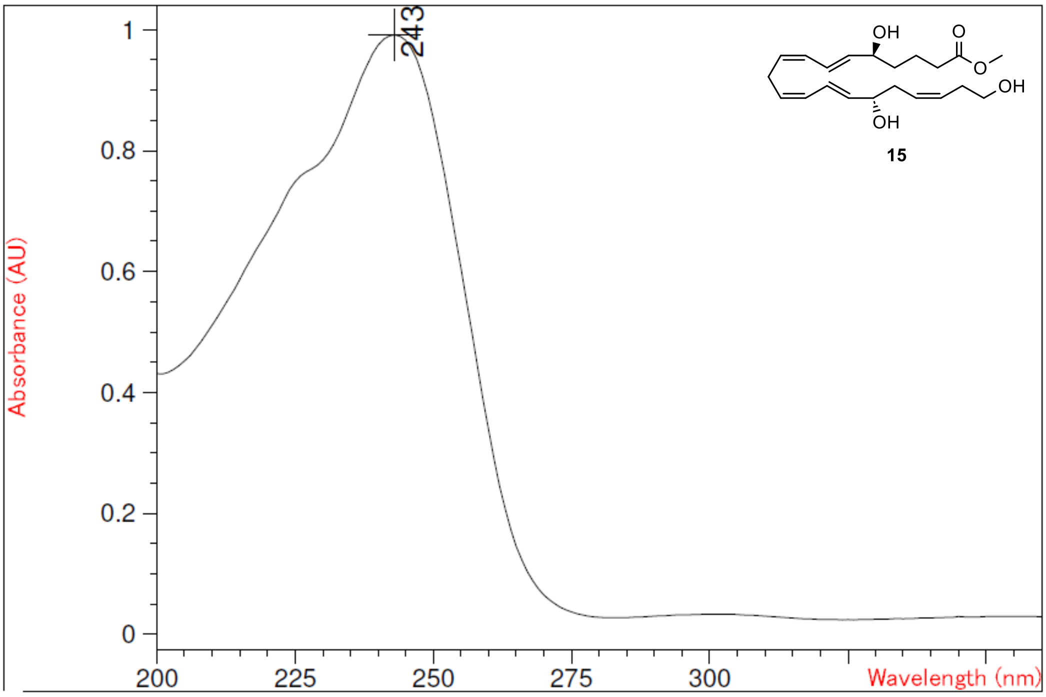

Figure S13 UV-Vis spectrum of 20-OH-RvE4 methyl ester (**15**).

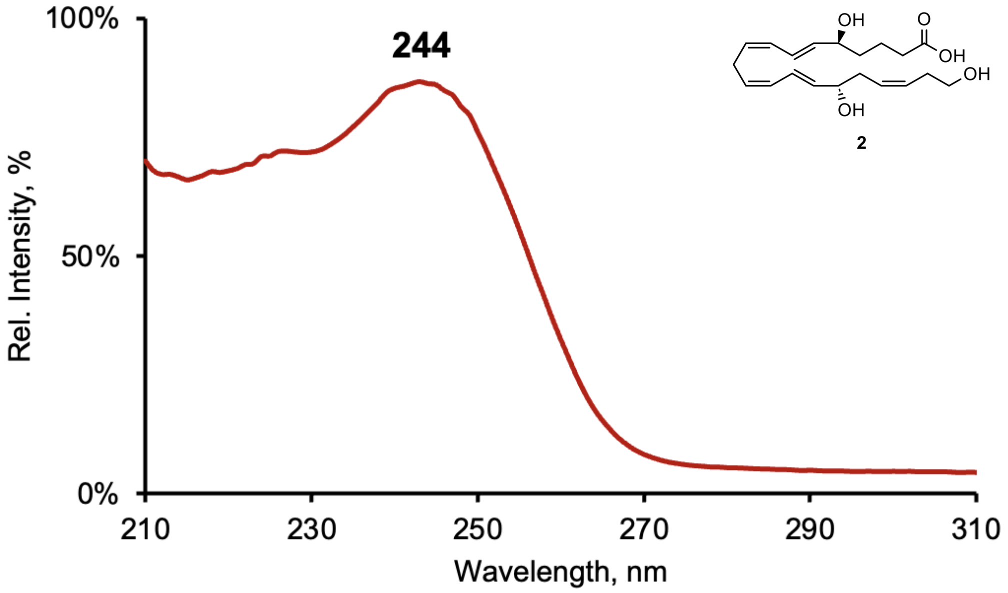

Figure S14 UV-Vis spectrum of 20-OH-RvE4 (**2**).

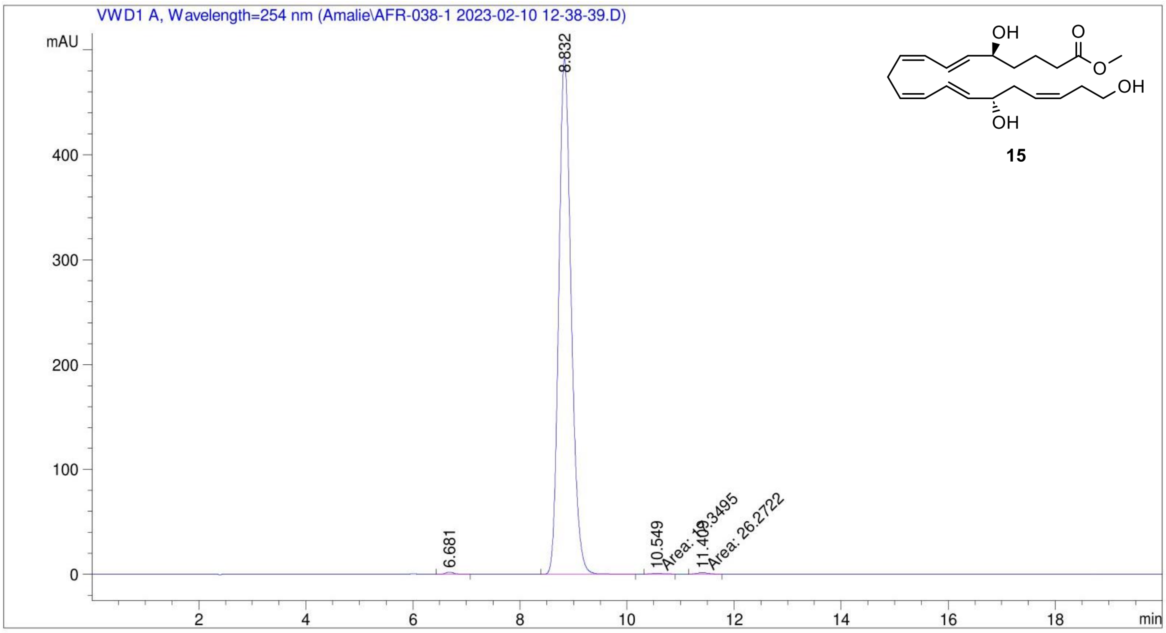

===
Area Percent Report
===

Sorted By : Signal
Multiplier : 1.0000
Dilution : 1.0000
Use Multiplier & Dilution Factor with ISTDs

Signal 1: VWD1 A, Wavelength=254 nm

Peak #	RetTime [min]	Type	Width [min]	Area [mAU*s]	Height [mAU]	Area %
1	6.681	BB	0.1853	25.81602	2.13224	0.3221
2	8.832	BB	0.2486	7944.28320	491.30557	99.1335
3	10.549	MM	0.3362	17.34955	8.60068e-1	0.2165
4	11.409	MM	0.2974	26.27220	1.47254	0.3278

Totals : 8013.72097 495.77042

Figure S15 HPLC spectrum of 20-OH-RvE4 methyl ester (**15**).

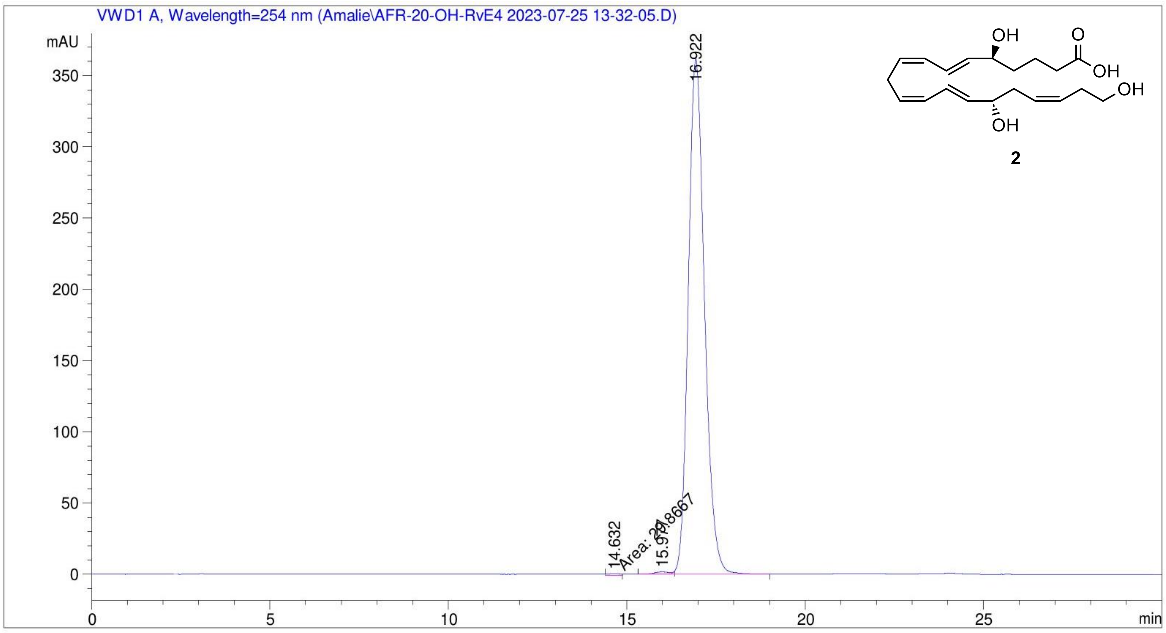

==

Area Percent Report

==

Sorted By : Signal
Multiplier : 1.0000
Dilution : 1.0000
Use Multiplier & Dilution Factor with ISTDs

Signal 1: VWD1 A, Wavelength=254 nm

Peak #	RetTime [min]	Type	Width [min]	Area [mAU*s]	Height [mAU]	Area %
1	14.632	MM	0.4446	29.86674	1.11974	0.2596
2	15.977	BV E	0.4032	46.15731	1.73728	0.4011
3	16.922	VB R	0.4882	1.14305e4	361.63113	99.3393
Totals :				1.15066e4	364.48815	

Figure S16 HPLC spectrum of 20-OH-RvE4 (**2**).

Acquisition Parameter

Source Type	ESI	Set Capillary	3500 V	Set Nebulizer	2.0 Bar
Focus	Not active	Set End Plate Offset	-500 V	Set Dry Heater	200 °C
Scan Begin	50 m/z	Set Charging Voltage	2000 V	Set Dry Gas	4.0 l/min
Scan End	1500 m/z	Set Corona	0 nA	Set Divert Valve	Waste
				Set APCI Heater	0 °C

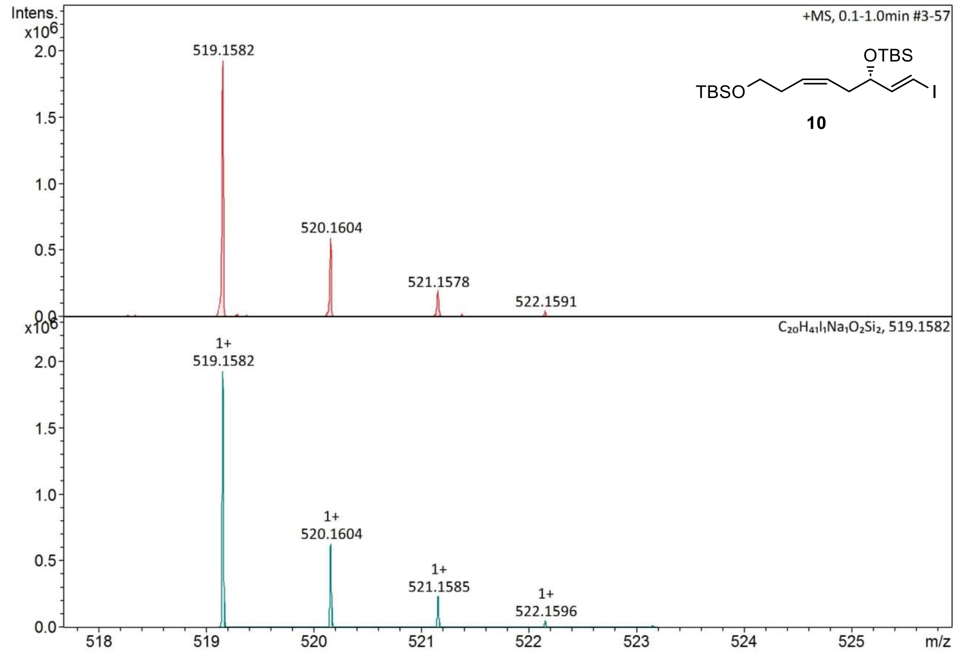

Meas. m/z	Ion Formula	m/z	err [ppm]
519.1582	C18H36IN6Si2	519.1579	-0.5
	C20H41INaO2Si2	519.1582	0.1

Figure S17 HRMS spectrum of compound **10**.

Acquisition Parameter

Source Type	ESI	Set Capillary	3500 V	Set Nebulizer	2.0 Bar
Focus	Not active	Set End Plate Offset	-500 V	Set Dry Heater	200 °C
Scan Begin	50 m/z	Set Charging Voltage	2000 V	Set Dry Gas	4.0 l/min
Scan End	1500 m/z	Set Corona	0 nA	Set Divert Valve	Waste
				Set APCI Heater	0 °C

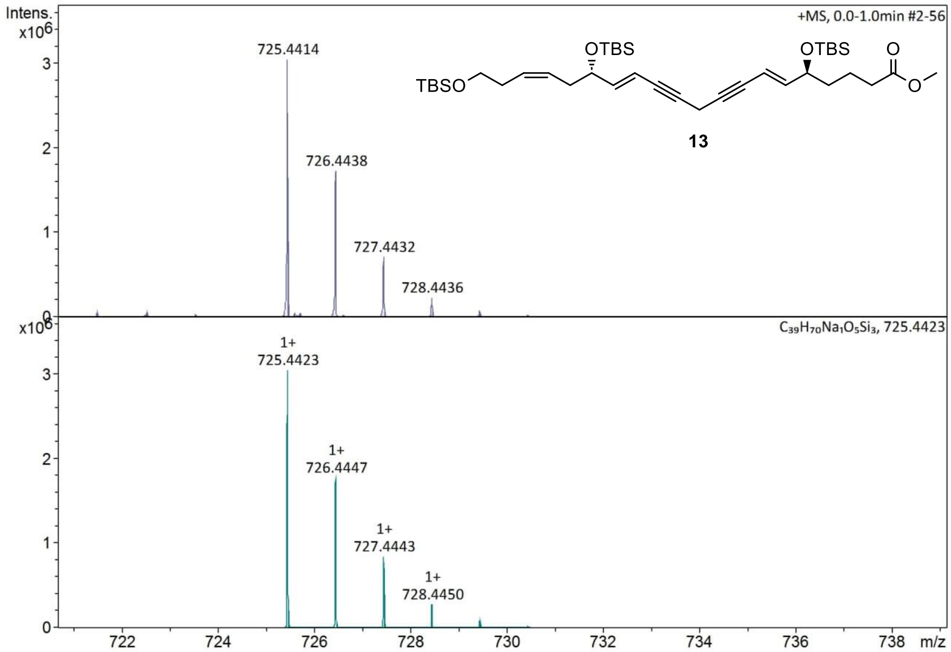

Meas. m/z	Ion Formula	m/z	err [ppm]
725.4414	C45H65O4Si2	725.4416	0.3
	C39H70NaO5Si3	725.4423	1.3

Figure S18 HRMS spectrum of compound **13**.

Acquisition Parameter

Source Type	ESI	Set Capillary	3500 V	Set Nebulizer	0.5 Bar
Focus	Not active	Set End Plate Offset	-500 V	Set Dry Heater	200 °C
Scan Begin	50 m/z	Set Charging Voltage	2000 V	Set Dry Gas	4.0 l/min
Scan End	1500 m/z	Set Corona	0 nA	Set Divert Valve	Waste
				Set APCI Heater	0 °C

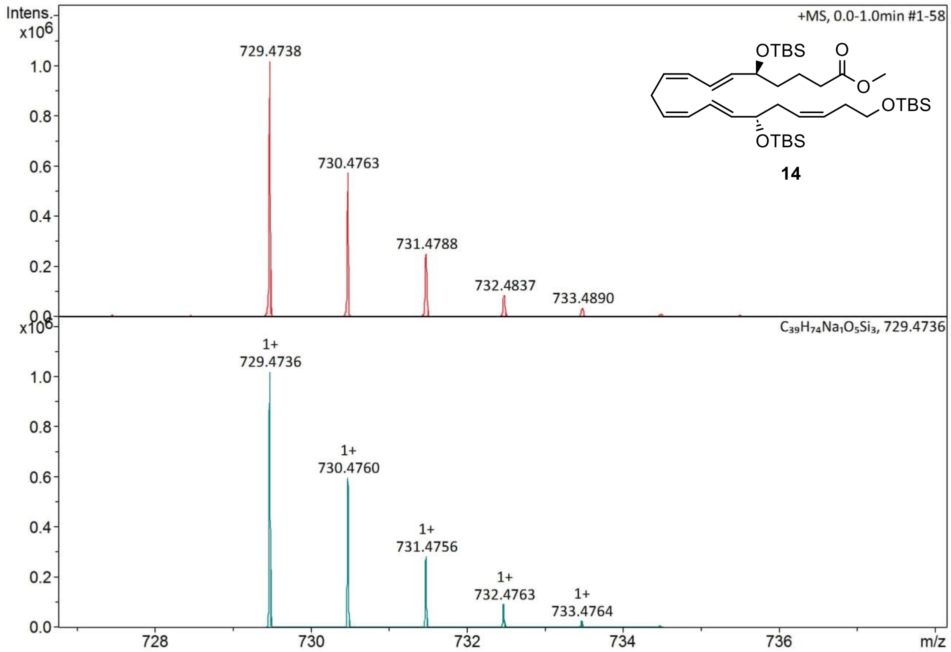

Meas. m/z	Ion Formula	m/z	err [ppm]
729.4738	C37H69N6O3Si3	729.4733	-0.6
	C39H74NaO5Si3	729.4736	-0.2

Figure S19 HRMS spectrum of compound **14**.

Acquisition Parameter

Source Type	ESI	Set Capillary	3500 V	Set Nebulizer	1.0 Bar
Focus	Not active	Set End Plate Offset	-500 V	Set Dry Heater	200 °C
Scan Begin	50 m/z	Set Charging Voltage	2000 V	Set Dry Gas	4.0 l/min
Scan End	1500 m/z	Set Corona	0 nA	Set Divert Valve	Waste
				Set APCI Heater	0 °C

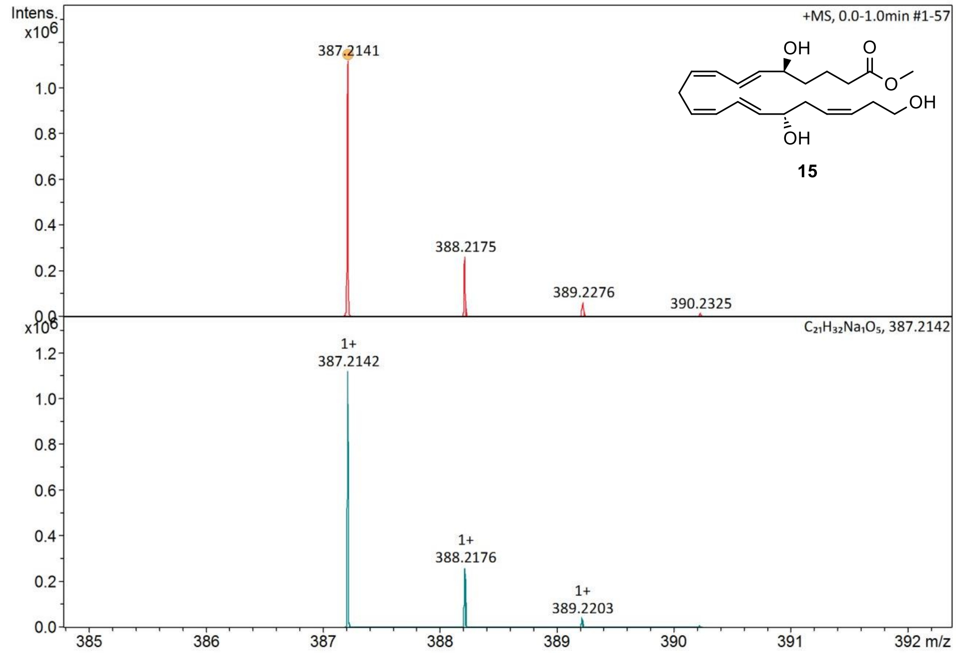

Meas. m/z	Ion Formula	m/z	err [ppm]
387.2141	C19H27N6O3	387.2139	-0.6
	C21H32NaO5	387.2142	0.1

Figure S20 HRMS spectrum of 20-OH-RvE4 methyl ester (**15**).

Acquisition Parameter

Source Type	ESI	Set Capillary	3500 V	Set Nebulizer	0.5 Bar
Focus	Not active	Set End Plate Offset	-500 V	Set Dry Heater	200 °C
Scan Begin	50 m/z	Set Charging Voltage	0 V	Set Dry Gas	4.0 l/min
Scan End	1500 m/z	Set Corona	0 nA	Set Divert Valve	Source
				Set APCI Heater	0 °C

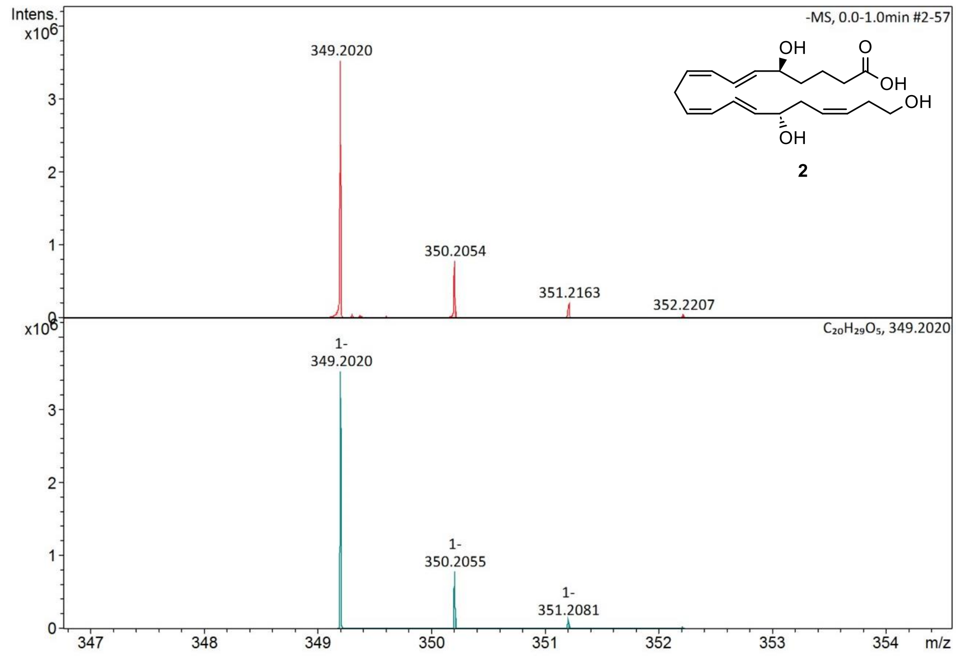

Meas. m/z	Ion Formula	m/z	err [ppm]
349.2020	C20H29O5	349.2020	0.1

Figure S21 HRMS spectrum of 20-OH-RvE4 (**2**).
